# Anaerobic methane oxidation inducing carbonate precipitation at abiogenic methane seeps in the Tuscan archipelago (Italy)

**DOI:** 10.1371/journal.pone.0207305

**Published:** 2018-12-19

**Authors:** Patrick Meister, Johanna Wiedling, Christian Lott, Wolfgang Bach, Hanna Kuhfuß, Gunter Wegener, Michael E. Böttcher, Christian Deusner, Anna Lichtschlag, Stefano M. Bernasconi, Miriam Weber

**Affiliations:** 1 Department of Geodynamics and Sedimentology, University of Vienna, Vienna, Austria; 2 HYDRA Marine Sciences GmbH, Sinzheim, Germany and HYDRA Field Station Elba, Italy; 3 Max Planck Institute for Marine Microbiology, Bremen, Germany; 4 MARUM–Center for Marine Environmental Research, University of Bremen, Bremen, Germany; 5 Geochemistry & Isotope Biogeochemistry Group, Leibniz Institute for Baltic Sea Research (IOW), Warnemünde, Germany; 6 GEOMAR Helmholtz Centre for Ocean Research, Kiel, Germany; 7 National Oceanography Centre, University of Southampton Water Front Campus, Southampton, United Kingdom; 8 Geological Institute, ETH Zürich, Zürich, Switzerland; Stockholms Universitet, SWEDEN

## Abstract

Seepage of methane (CH_4_) on land and in the sea may significantly affect Earth’s biogeochemical cycles. However processes of CH_4_ generation and consumption, both abiotic and microbial, are not always clear. We provide new geochemical and isotope data to evaluate if a recently discovered CH_4_ seepage from the shallow seafloor close to the Island of Elba (Tuscany) and two small islands nearby are derived from abiogenic or biogenic sources and whether carbonate encrusted vents are the result of microbial or abiotic processes. Emission of gas bubbles (predominantly CH_4_) from unlithified sands was observed at seven spots in an area of 100 m^2^ at Pomonte (Island of Elba), with a total rate of 234 ml m^-2^ d^-1^. The measured carbon isotope values of CH_4_ of around -18‰ (VPDB) in combination with the measured δ^2^H value of -120‰ (VSMOW) and the inverse correlation of δ^13^C-value with carbon number of hydrocarbon gases are characteristic for sites of CH_4_ formation through abiogenic processes, specifically abiogenic formation of CH_4_ via reduction of CO_2_ by H_2_. The H_2_ for methanogenesis likely derives from ophiolitic host rock within the Ligurian accretionary prism. The lack of hydrothermal activity allows CH_4_ gas to become decoupled from the stagnant aqueous phase. Hence no hyperalkaline fluid is currently released at the vent sites. Within the seep area a decrease in porewater sulphate concentrations by ca. 5 mmol/l relative to seawater and a concomitant increase in sulphide and dissolved inorganic carbon (DIC) indicate substantial activity of sulphate-dependent anaerobic oxidation of methane (AOM). In absence of any other dissimilatory pathway, the δ^13^C-values between -17 and -5‰ in dissolved inorganic carbon and aragonite cements suggest that the inorganic carbon is largely derived from CH_4_. The formation of seep carbonates is thus microbially induced via anaerobic oxidation of abiotic CH_4_.

## Introduction

Seepage represents an important route of transport for methane (CH_4_) and other hydrocarbon gases from Earth’s subsurface to the oceans and the atmosphere. When CH_4_ comes in contact with seawater, it provides energy to drive chemosynthetic microbial activity and a zone of anaerobic oxidation of methane (AOM) coupled to sulphate reduction in marine porewater gets established. Much of the dissolved methane is oxidized in this zone, but the rise of CH_4_ bubbles can be vigorous enough to by-pass the AOM trap and shuttle methane into the overlying water column (e.g. [1]Judd, 2004; [2]Greinert et al. 2006). Escaping CH_4_ can then contribute to the atmospheric CH_4_-pool where it acts as a strong greenhouse gas. Finding the locations of CH_4_-seepage, tracing the origin of the CH_4_ and identifying abiotic vs. biotic processes occurring at CH_4_ seeps is therefore of fundamental importance for understanding the global carbon cycle and its interaction with the biosphere.

Most CH_4_ released by seepage at the seafloor is derived from the decomposition of organic matter. Methane is produced either by microbial methanogenesis or by thermal breakdown of larger organic molecules at higher temperatures. Both types of CH_4_ generation have been described in many studies from modern and fossil seep locations (e.g. microbial CH_4_: [3]Roberts and Aharon, 1994; [4]Birgel et al., 2011; [5]Natalicchio et al., 2012; vs. thermogenic CH_4_: [6]Hovland and Judd, 1988; [7]Brooks et al., 1986; [8]Jessen et al., 2011). Thermogenic CH_4_ is generally less depleted in ^13^C compared to microbial CH_4_ ([9][10]Schoell, 1980, 1988; [11]Whiticar, 1999), as during the thermogenic breakdown of hydrocarbons, CH_4_ largely inherits the isotopic composition of the organic matter, modified only by minor kinetic fractionation ([12]Berner et al., 1995; [13]Fiebig et al., 2007). In contrast, microbial CH_4_ can be very strongly depleted in ^13^C (see below), due to kinetic isotope fractionation by microbial enzymatic pathways of methanogenesis (e.g. [14]Claypool and Kaplan, 1974). Yet another type of CH_4_ seep exists where CH_4_ forms abiotically in crystalline basement (e.g. [15]Berndt, 1996; [16]Sherwood Lollar et al., 1993; [17]Etiope and Sherwood Lollar, 2013). Methane can form abiotically from inorganic carbon via a Fischer-Tropsch-type reaction ([18]Lancet and Anders, 1970). Another formation mechanism for methane is the Sabatier reaction:

$$4\;H_{2}+{CO}_{2}\to {CH}_{4}+2H_{2}O$$ (1)

Regardless of the reaction pathway, the abiogenic formation of CH_4_ is fuelled by dihydrogen (H_2_), which forms as a result of subsurface water-rock interactions, such as serpentinization of ultramafic rocks (e.g. [19]Sleep et al., 2004; [20]McCollom and Bach, 2009). Methane can only form from CO_2_-reduction if H_2_ is produced in large-enough quantities. This quantity of H_2_, or–more specifically–the H_2_-activity of the interacting fluid critically depends on the fluid-mineral equilibria in the system, which is dependent on pressure, temperature, and system composition. The highest H_2_-activities known in natural systems develop during serpentinization of olivine ((Mg,Fe)_2_SiO_4_) in ultramafic rocks to serpentine, brucite, and magnetite:

$$3\;{Fe}_{2}SiO_{4}+2\;H_{2}O\to 2\;{Fe}_{3}O_{4}+3\;SiO_{2}(aq)+2\;H_{2}$$ (2)

where Fe_2_SiO_4_ is fayalite (Fe-olivine) and Fe_3_O_4_ is magnetite, and:

$$2\;{Mg}_{2}SiO_{4}+3\;H_{2}O\to {Mg}_{3}{Si}_{2}O_{5}{\left(OH\right)}_{4}+Mg{\left(OH\right)}_{2}$$ (3)

where Mg_2_SiO_4_ is forsterite (Mg-olivine), Mg_3_Si_2_O_5_(OH)_4_ is serpentine, and Mg(OH)_2_ is brucite.

As silica activity is buffered to low values by the presence of serpentine and brucite that form from hydration of the forsterite component in olivine (Eq 3), the equilibrium activity of dihydrogen is exceedingly high (note the power of 3 for aqueous silica in the mass action equation for Eq 2). The overall reaction is thermodynamically favoured at temperatures below about 350°C ([20]McCollom and Bach, 2009) but the abiotic kinetics are sluggish at low temperatures ([21]McCollom, 2016). Abiogenic CH_4_ is predominantly released from hydrothermal vent systems along oceanic spreading centres, such as the Lost City Hydrothermal Field in the central Atlantic ([22][23]Kelley et al., 2001, 2005; [24][25]Früh-Green et al., 2003, 2007), and many other sites along oceanic spreading centres (e.g. [26]Welhan, 1988; [27]McDermott et al., 2015). In contrast to microbially derived CH_4_, which usually has δ^13^C values < -60‰ ([9][10]Schoell, 1980, 1988; [11]Whiticar, 1999), rarely up to -47‰ ([28]Bradley and Summons, 2010), abiogenic CH_4_ is commonly less depleted in ^13^C, with δ^13^C-values ranging from -40 to -5‰ ([29]Hoefs et al., 2018; [30]D’Alessandro et al., 2018). The origin of methane in hydrothermal vent fluids is often unclear, but abiogenic production of methane from CO_2_-reduction is thermodynamically favourable only in fluids from ultramafic-hosted systems ([31]McCollom and Seewald, 2007). This methane in ultramafic-hosted seafloor hydrothermal sites is generally enriched in ^13^C (δ^13^C of -15‰ to -5‰; [32]Konn et al., 2015) compared to microbial or thermogenic CH_4_. Since the isotope signatures of the CH_4_ are often preserved in the seep carbonates and therefore in the geological record, these carbonates may be used as archives of past CH_4_ seepage. Strongly negative δ^13^C-values of calcified pavements and circular or pipe-like conduits that form as a result of TA increase during AOM (e.g. [33]Stakes et al., 1999; [34]Aiello et al., 2001; [35]Aiello, 2005; [5]Natalicchio et al., 2013) are commonly observed related to microbial or thermogenic CH_4_ seeps. In contrast, abundant carbonate precipitates with chimneys of up to sixty metres high, as observed at Lost City-type hydrothermal vents ([23]Kelley et al. 2005), are not induced by AOM but form due to mixing of seawater with alkaline fluids from serpentinization. These carbonates show δ^13^C values in the range of carbonate veins formed by low-temperature reactions of serpentinites with seawater, i.e. around 0‰ (e.g. [36]Bach et al., 2011).

Abiogenic CH_4_ seeps related to serpentinization also occur in shallow water (e.g. Bay of Prony, New Caledonia; [37]Monnin et al., 2014) or even on land (e.g. Franciscan Complex, California: [38]Barnes and O’Neil, 1969; [39]Morrill et al., 2013; Voltri, Italy: [40]Schwarzenbach et al., 2013; [41]Boschetti et al., 2013; [42]Chavagnac et al., 2013). Seep carbonates at such sites likely result from hyperalkalinity of the waters (from serpentinization) and with exposure to atmospheric CO_2_ rather than from CH_4_ oxidation. Thus, they show the isotopic composition of the ambient inorganic carbon (e.g. [43]Meister et al., 2011a). Other methane seeps on land have thermogenic or microbial sources, which can be distinguished from serpentinization-related sources based on methane/ethane ratios and the C- and H-isotopic composition of methane ([17]Etiope et al., 2013). Geothermally active areas can also feature methane seepage, although those seep gases are also rich in CO_2_ ([13]Fiebig et al., 2007).

**Fig 1:**
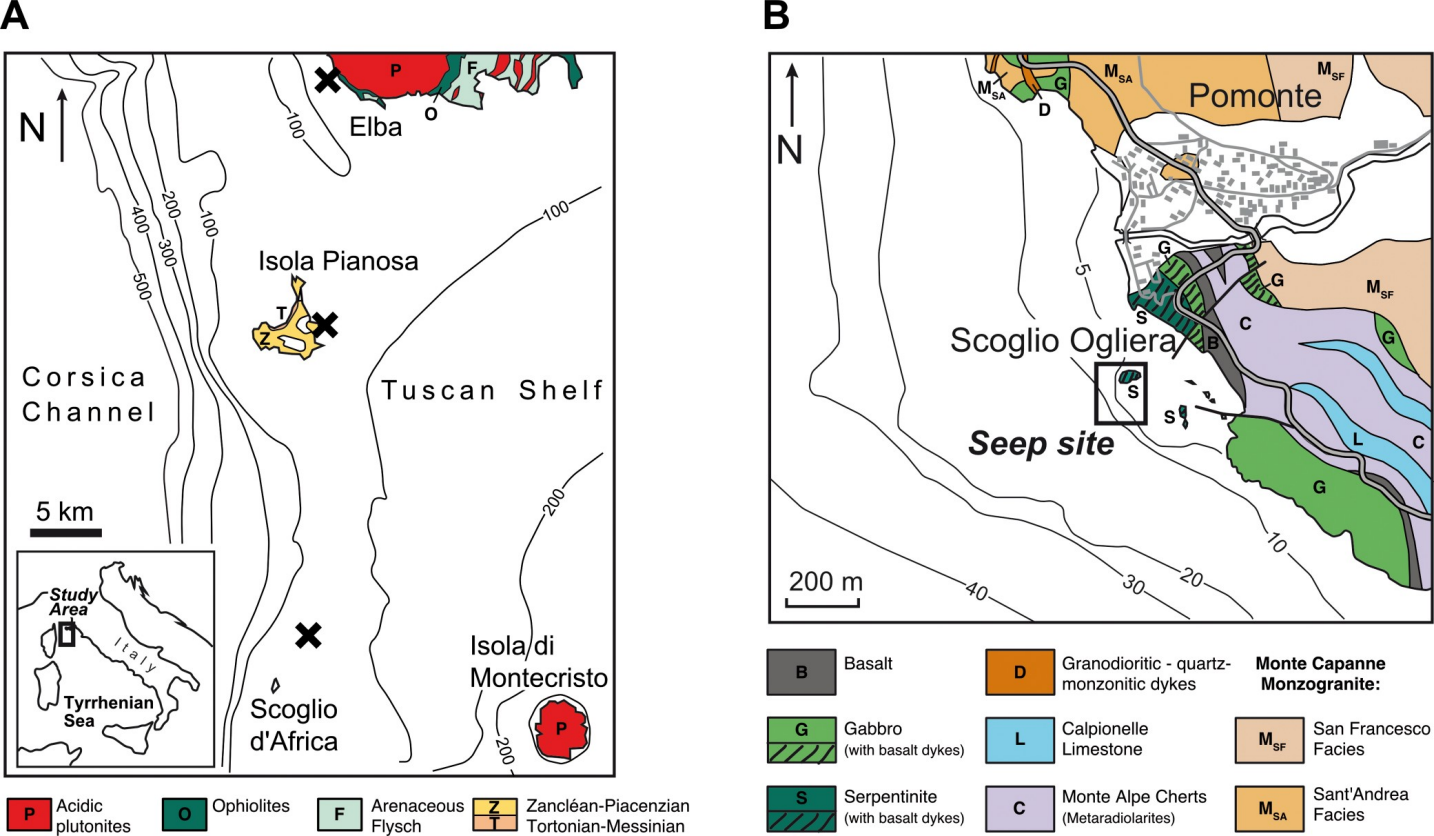
Sampling location. (A) Map of the Tyrrhenian Sea showing the locations of hydrocarbon gas seeps (crosses) near Pomonte (Island of Elba), the island of Pianosa and the Scoglio d’Africa. (B) Close-up of the area and geology around the CH_4_ seep near Pomonte. Based on the geological map of Elba Island [45](Principi et al., 2008).

Here we report the results of a geochemical study of a number of gas seeps that occur in shallow water near the coast of Pomonte (Island of Elba), the Island of Pianosa and the Scoglio d’Africa (Fig 1). These sites have been investigated by divers of the HYDRA field station since 1995 and were recently analysed in a molecular-biological study ([44]Ruff et al., 2016). These sites have been noticed due to emanating CH_4_ bubbles and areas of discoloured sediment containing semi-lithified carbonate crusts. So far, the source of CH_4_ has not been identified, and it is hence unclear whether it is produced by microbial methanogenesis, thermogenesis, or abiotic (volcanic or hydrothermal) source. It is also not clear, how the emanating CH_4_ affects microbial activity at the vent sites and whether the carbonate precipitates observed at the CH_4_ emission spots are a result of this activity.

The goal of this study is to resolve the origin of CH_4_ and to identify biogeochemical processes occurring at the gas emission spots. Our aim is also to understand how these processes are reflected in the formation of seep carbonates. Therefore, we describe and quantify gas seepage and present new geochemical and isotope data. Gas compositions and stable isotope signatures were determined and serve as indicators of possible origins of the CH_4_. Gas data are compared with porewater chemistry and, combined with radiotracer incubation experiments revealing AOM activity, interpreted with respect to biogeochemical processes occurring at the seep sites. The carbonate cements at the Pomonte seeps were examined using microscopic and x-ray diffraction techniques. Carbon and oxygen isotope compositions of the carbonates were measured to constrain their origin and temperature of precipitation. Based on these findings a conceptual model for the processes leading to CH_4_ seepage at the Pomonte site is presented.

## Geological setting and study site

The main lithologic units of the Island of Elba represent deep-sea sediments and ophiolites of the Mesozoic Ligurian ocean. During the Late Miocene (8.5–6.85 Ma ago) the Tyrrhenian Sea opened as a back-arc basin between the Apennine and Corsica/Sardinia ([46]Frisch et al., 2008). This episode resulted in a 900 m deep basin between Corsica and the Tuscan Shelf, on which Elba is located (Fig 1A). This post-collisional extension of the Apennine has caused lithospheric thinning and related melting of the asthenosphere. These mantle melts interacted with the rifted crust to produce varied intermediate to felsic intrusive bodies (e.g. [47]Collettini et al., 2006). Today, the northern Tyrrhenian Sea is characterized by relatively thin crust ([48]Scarascia et al., 1994) on the order of 22 km and a shallow asthenosphere at 50 km depth. In areas of the Tyrrhenian Sea and Western Apennines region where the magmatic intrusions are most recent, heat flow is high, especially in Tuscany ([49]Scrocca et al., 2003). Geothermal heat fluxes of up to 1000 mW·m^-2^ ([50]Smith et al., 2011) have been attributed to young plutonic intrusions in that region. This thermal energy is being commercially used at the Lardarello geothermal power station on the Tuscan mainland ([51]Minissale et al., 1997; [52]Lund, 2004).

In Elba, these intrusions were emplaced in a sinistral pull-apart void within the Jurassic Ligurian oceanic complex ([53]Bortolotti et al., 2001; [46]Frisch et al., 2008) about 6–7 million years ago ([54]Bouillin et al., 1993; [55]Dini et al., 2002). The Monte Capanne pluton (Fig 1A), in the western part of Elba, is the westernmost and oldest magmatic body of the Tuscan magmatic province. Syn- to post-intrusive are east-vergent low-angle detachment faults ([50]Smith et al., 2011) along which the pluton was uplifted and denuded. Today, heat flow in western Elba is not elevated, unlike in the eastern part of the Tuscan magmatic province. The Monte Capanne pluton intruded Jurassic to Cretaceous ophiolitic units; sets of porphyric dike swarms cross cut the pluton and host lithologies ([55]Dini et al., 2002).

The western coast of Elba adjacent to the Pomonte seep sites is dominated by monzogranitic rocks of the Monte Capanne intrusion ([45]Principi et al., 2008) with downfaulted slabs of the intruded ophiolitic unit, consisting of peridotite (lherzolite and harzburgite; [56]Viti and Mellini, 1998, [45]Principi et al., 2008), gabbro, pillow basalt, and sedimentary breccia, overlain by cherts and limestones ([57]Keller and Pialli, 1990). These rocks are affected by contact metamorphism with skarn formation ([58]Rossetti and Tecce, 2008). Along the pluton's southwestern rim, between the villages Chiessi and Fetovaia, serpentinite and turmaline-bearing aplite dykes in metamorphosed radiolarite are found. Further south, the peninsula of Fetovaia is composed of coarse gabbro cross-cut by pegmatitic dykes ([46]Frisch et al., 2008). The Scoglio Ogliera, a small rock island adjacent to the sampling site (Fig 1B), consists of gabbro while the rocks cropping out on shore show the entire ophiolite sequence from serpentinite, gabbro, basalt to radiolarite, calpionella limestone and argille a palombini ([45]Principi et al., 2008). The only outcrop of limestone in the area is a part of a pre- and post-Messinian sediment sequence as part of the Elba-Pianosa ridge on the island of Pianosa. Scoglio d’Africa sits on top of a fault-bounded horst structure consisting of Triassic-Liassic limestone based on seismics ([59]Cornamusini et al., 2002).

### Gas seeps in the Tyrrhenian Sea and the Italian peninsula

Many seeps and hydrothermal systems producing various amounts of CH_4_ are known from the areas surrounding the Tyrrhenian Sea and along the Italian peninsula. Probably all types of CH_4_ seeps occur, depending on the tectonomagmatic and sedimentary setting. [60]Tassi et al. (2012) compiled geochemical data from 87 locations, which can be subdivided into four regional domains based on the geological setting and geochemical properties of the emanating fluids: (1) The Latio-Umbria region and Tuscan Magmatic Province (e.g. Larderello and Mt. Amiata hydrothermal springs; [61]Gherardi et al., 2005) are grouped as “Tyrrhenian” domain that is largely associated with the backarc extensional regime. (2) Admixture of CH_4_ with volcanic gas at Vesuvio, Campi Flegrei, Panarea and Pantelleria result from interaction with calc-alkaline rocks ([60]Tassi et al., 2012). (3) Mud volcanoes occurring frequently along the Appennine and Adriatic coast are releasing microbial or thermogenic CH_4_ from a thick pile of sedimentary nappes along the Adriatic thrust front. (4) Abiogenic CH_4_ is released from hyperalkaline springs related to serpentinization of Ligurian ophiolites near Genova ([41]Boschetti et al., 2013).

## Material and methods

### Site investigation and sampling

**Fig 2:**
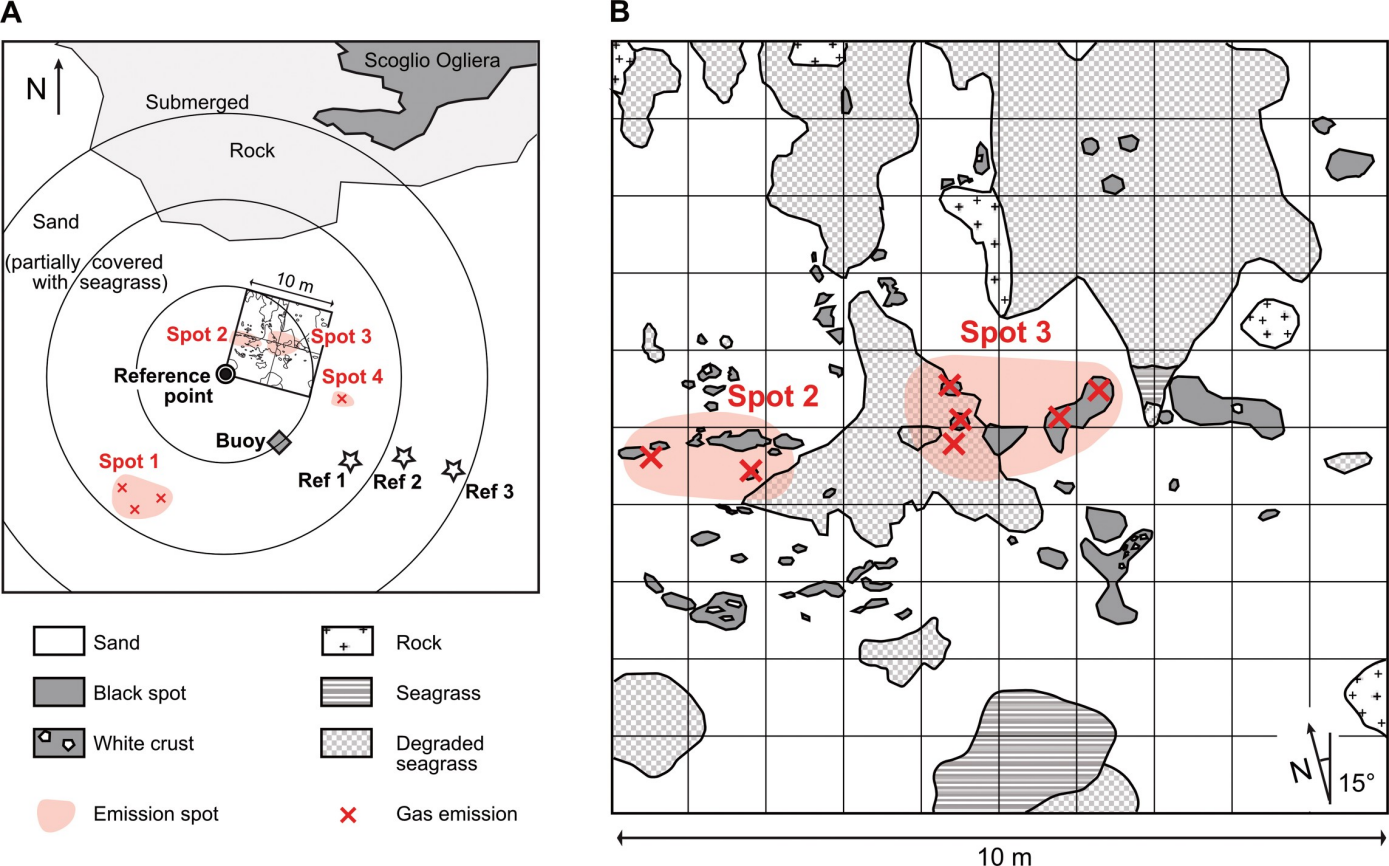
Seafloor map of methane seeps. (A) Seafloor map of the Pomonte seep site. The coordinate of the reference point is 42° 44.628' N, 010° 07.094' E. (B) Close up of main 100 m^2^ area, where several gas emission spots were documented, although their actual position changes over time by several centimetres.

Three marine gas seep sites were studied: (i) located off the coast of the village Pomonte, southwest of the Capanne pluton, (ii) approximately 14 km south of Elba at the east coast of the Island of Pianosa (access authorized by the administration of the Parco Nazionale dell'Arcipelago Toscano; permit no. 2930/2017), and (iii) 40 km south of Elba near the islet of Scoglio d’Africa (Fig 1A). The Pomonte site is about 200 m from shore (Fig 1B) and covers about 1000 m^2^ (42° 44.628' N, 010° 07.094' E; Fig 2).

The seep area at Pomonte was investigated by SCUBA diving from 2008 to 2011 and in 2015. The area was mapped in detail using a 10 × 10 m line grid (Fig 2) and searched for gas emissions by divers in concentric circles at 10, 20 and 30 m distance. Water temperature was measured using a handheld thermometer. For sampling, three emission spots and three reference sites without visible gas emission were chosen. From the emission spots gas was sampled *in situ* with a funnel into Exetainer glass vials (Labco, UK) and kept at room temperature until analysis. The emission rate was determined as volume per time by capturing gas into a scaled cylinder and calculated to an areal rate (ml m^-2^ d^-1^). Carbonate precipitates larger than one cubic centimetre in size were collected using a hand-held underwater suction pump, then washed with tap water and air-dried. Bulk sediment was recovered with 70-cm long PVC coring tubes (diameter 4.5 cm). On land, the sediment cores were sliced into 2- or 10-cm thick layers. Samples for geochemical and mineralogical analyses were stored at -20°C until measurement. Prior to analysis the sediment was freeze-dried and ground with agate mortar and pestle.

Porewater was sampled at the seafloor by penetrating the emission spot with a stainless steel lance (1 m length, 2 ml dead volume) and attached plastic syringes in October 2009, April 2010 and April 2011. Samples were taken every 10 cm, down to a maximum depth of 60 cm. The dead space was flushed before each new sample to avoid contamination. Samples for sulphate, chloride and sulphide analyses were fixed under water with 5% zinc acetate, which was pre-filled in the syringes. On land, water samples for DIC measurements were filled headspace-free into 2 ml borosilicate glass vials and fixed with 0.25 M HgCl_2_. Samples for cation analysis were filled into pre-cleaned polyethylene vials and acidified with nitric acid (70%) to a final concentration of 2%. For dissolved CH_4_ measurements, 16 ml of porewater were added to 50 ml glass bottles containing 20 ml of 2.5% NaOH solution. The bottles were sealed with butyl rubber stoppers and vigorously shaken. The headspace was analysed for CH_4_ concentrations. All porewater samples were stored at 4°C.

### Sediment analysis

Permeability was measured by percolating water through 20 and 50 cm long cores and measuring the flux rate ([62]Lambe and Whitman, 1969). Porosity was determined by weight loss after drying of fresh wet sediment ([63]Higgins and Thiel, 1988). Grain size distribution was analysed with a series of sieves in a Rentsch vibraxer (Type S-S, 23800) and reported according to the Wentworth-Scale ([64]Wentworth, 1922). Sediment cores were microscopically screened for carbonate precipitates in sub-samples of 5 g sediment every 5 cm depth. Photographs of the thin sections were taken under cross-polarized light using a Leica DM2700 P microscope equipped with a Leica MC170 HD camera. The microscopic structures of precipitated minerals were analysed by a ZEISS SUPRA40 scanning electron microscope equipped with an Oxford PentaFETx3 energy dispersive x-ray detector.

The mineralogy was determined by a Philips XPERT pro powder X-ray diffractometer, using CuKα radiation and scanning angles (2θ) from 3 to 85°. The proportions of different minerals were estimated from integrated peak areas. Ca/Mg ratios of the precipitates were calculated using the calibration given by [65]Lumsden (1979). Total carbon (TC) content was determined with a Carlo Erba NA-1500 CNS analyser using in-house standard (DAN1). Total inorganic carbon (TIC) was analysed with an UIC Inc. CM 5012 CO_2_ coulometer equipped with a CM 5130 acidification module. Precisions (2σ) were 0.2 wt % for TC and 0.1 wt % for TIC. Total organic carbon (TOC) was calculated from the difference between TC and TIC.

The amounts of acid volatile sulphide (AVS) and chromium reducible sulphur (CRS) within the sediment were analysed according to [66]Fossing and Jørgensen (1989). Five grams of wet sediment were sequentially distilled using 6 N HCl for AVS and reduced Cr solution for CRS, and each fraction was recovered in a zinc acetate trap. Sulfide concentrations were measured by the method of [67]Cline (1969) as described below.

Carbon and oxygen isotopes in micro-drilled and powdered samples were analysed with a Finnigan MAT 251 mass spectrometer coupled to an automated Kiel acidification device. The analytical precision of the mass spectrometer was ± 0.05‰ for δ^13^C, and ± 0.07‰ for δ^18^O. Powdered Solnhofener Plattenkalk calibrated against the NBS standard was used as a working standard and the δ^13^C and δ^18^O values of the carbonates are reported relative to the Vienna Peedee Belemnite Standard (VPDB). δ^18^O data of the precipitates and the adjacent porewater were used for the calculation of the isotope equilibrium and the crystallization temperature using the calibration equation given by [68]Kim et al. (2007).

### Porewater analysis

Sulphate and chloride concentrations were measured by non-suppressed anion exchange chromatography (Waters IC-Pak anion exchange column, Waters 430 conductivity detector). Total dissolved sulphide concentrations were measured, using the diamine method ([67]Cline, 1969). DIC concentrations were measured via flow injection by conductivity detection (Van Waters and Rogers Scientific, model 1054) using 30 mmol/l HCl and 10 mmol/l NaOH as eluents ([69]Hall and Aller, 1992). δ^13^C_DIC_ was measured with a Finnigan MAT 252 mass spectrometer connected to a GasBench. δ^18^O_H2O_ was determined by equilibration with CO_2_ using an automated Isoprep-18 equilibration device coupled to a Micromass Optima mass spectrometer. δ^18^O_H2O_ values are reported relative to Vienna standard mean ocean water (VSMOW).

Total alkalinity (TA) in combination with pH was measured with the method of end-point titration (modified after [70]Van den Berg and Rogers, 1987) using a pH-meter with a temperature probe (GPRT 1400 A, GREISINGER electronic GmbH) and 0.1 M HCl. Metal analyses in the porewaters were carried out at Leibniz IOW by ICP-OES (iCAP 6300 Duo, Thermo Fisher) after appropriate dilution. Accuracy and precision of the analyses were controlled by replicate measurements of a certified CASS seawater standard, as described by [71]Kowalski et al. (2012), and found to be better than ±3 and ±6%, respectively.

Methane dissolved in porewater was analysed at the Max Planck Institute for Marine Microbiology (Bremen) from alkalized headspace vials (2.5% NaOH) using a Hewlett Packard 5890A gas chromatograph (Hewlett Packard, Palo Alto, CA, U.S.A.) equipped with a packed stainless steel Porapak-Q column (6 ft, 0.125 in, 80/100 mesh; Agilent Technologies, Santa Clara, CA, USA) and a flame ionization detector. Helium was used as carrier gas at a flow rate of 2 ml min^−1^ and 40°C.

Based on the available porewater chemistry, aragonite saturation indices (SI = log IAP–log Ks; with IAP = ion activity product and Ks = solubility product) were calculated by the program PhreeqC ([72]Parkhurst and Appelo, 2013) using the PhreeqC.dat database. No significant differences in the calculated SI were observed when the Pitzer.dat database was used. Thermodynamic calculations were conducted using SUPCRT92 ([73]Johnson et al., 1992).

### Gas analysis

Gas compositions were analysed by the same method as described above. δ^3^C_CH4_ was measured by coupling a Trace GC ultra gas chromatograph via a GC combustion interface (Thermo Surveyor) to a mass spectrometer (Thermo Finnigan Delta Plus XP).

Additional gas samples from gas emission spot 1 were analysed for δ^13^C and δ^2^H in CH_4_ at Imprint Analytics GmbH (Neutal, Austria) using a gas chromatograph (Shimadzu GC 2010Plus, Shimadzu Corp., Kyoto, Japan) coupled to a stable isotope ratio mass spectrometer (Nu Horizon; Nu Instruments Limited, Wrexham, UK). Gas samples were directly injected by an AOC5000 Autosampler (CTC Analytics, Zwingen, Switzerland) from the Labco Exetainers into the S/Sl Injector of the GC. The CH_4_ was separated from other gases in a Q-Plot GC column (Supelco, Bellefonte USA) at 35°C (isothermal). The separated analytes were identified in a quadrupole mass spectrometer (Shimadzu GCMS-QP2010Ultra). Methane was oxidized to CO_2_ for the δ^13^C analyses in an oxidation oven filled with oxidized Ni and Pt wires at a temperature of 1040°C. For the δ^2^H analysis, CH_4_ was pyrolysed at 1400°C to H_2_ in a ceramic tube (Hekatech, Wegberg, Germany). As reference material hexane vapour with a known δ^13^C and δ^2^H composition was used. δ^13^C is reported in ‰ VPDB, δ^2^H in ‰ SMOW.

### Incubation experiments

Samples from 20–25, 40–45 and 60–65 cm sediment depth were collected in 500 ml sampling jars from emission and reference spots. To measure potential metabolic rates, replicates (n = 15 for each sample) of 2 g of wet-weight sediment mixed with anoxic seawater medium containing 28 mmol/l of sulphate ([74]Widdel and Bak, 1992) were incubated in 5 ml Hungate tubes. Of these a replicate series (n = 5) was equilibrated with CH_4_ (1 atm ~ 1.5 mM). A control series without CH_4_ (n = 5) was conducted to trace rates of background CH_4_-independent sulphate reduction (SR). No substrate besides CH_4_ was added to any of the incubations. Rates of total and CH_4_-independent SR were determined from these replicate incubations. After equilibration, ^35^SO_4_^2-^ tracer (80–100 kBq dissolved in 20 μl water) was injected into the Hungate tubes through a butyl rubber septum. Samples were incubated at room temperature for 3.25 days. To determine the SR rate, 1 ml of each sample was transferred into ZnCl (5%). Samples were processed as described by [75]Kallmeyer et al. (2004) and activities of total reduced inorganic carbon and sulphate were determined by scintillation counting (scintillation mixture; Ultima Gold, Perkin Elmer, Waltham, MA, USA; scintillation counter; 2900TR LSA; Packard Waltham, MA, USA). Rates of sulphate reduction were calculated with the Eq (4):

$$R_{SR}=[{SO}_{4}^{2-}]∙\frac{\left[{{}^{35}SO}_{4}^{2-}\right]}{\left[TRIS+{{}^{35}SO}_{4}^{2-}\right]}∙\frac{1}{t}∙1.06$$ (4)

where TRIS is the total reduced inorganic sulphur. The factor 1.06 corrects for the expected isotopic fractionation ([76]Jørgensen and Fenchel, 1974), t is time.

Rates of CH_4_ oxidation were measured in parallel in ^14^CH_4_ tracer incubations (15 kBq dissolved in 50 μl water; n = 5). The incubations were stopped by transferring the cultures into bottles with 25 ml of sodium hydroxide (2.5% w/v). The activities of ^14^CH_4_ and ^14^CO_2_ were measured via scintillation counting following protocols of [77]Treude et al. (2003). Methane concentrations were measured using gas chromatography (Focus GC, Thermo Scientific). Rates of AOM were calculated following Eq (5):

$$R_{AOM}=[{CH}_{4}]∙\frac{\left[{}^{14}{CO}_{2}\right]}{\left[{}^{14}{CO}_{2}+{}^{14}{CH}_{4}\right]}∙\frac{1}{t}$$ (5)

All rates were converted to μmol per litre of porewater per day assuming a density of the incubated sediment of 2.2 g cm^-3^ and using the measured porosity of 43%.

## Results

### Occurrence and distribution of methane seeps in the Tuscan Archipelago

**Fig 3:**
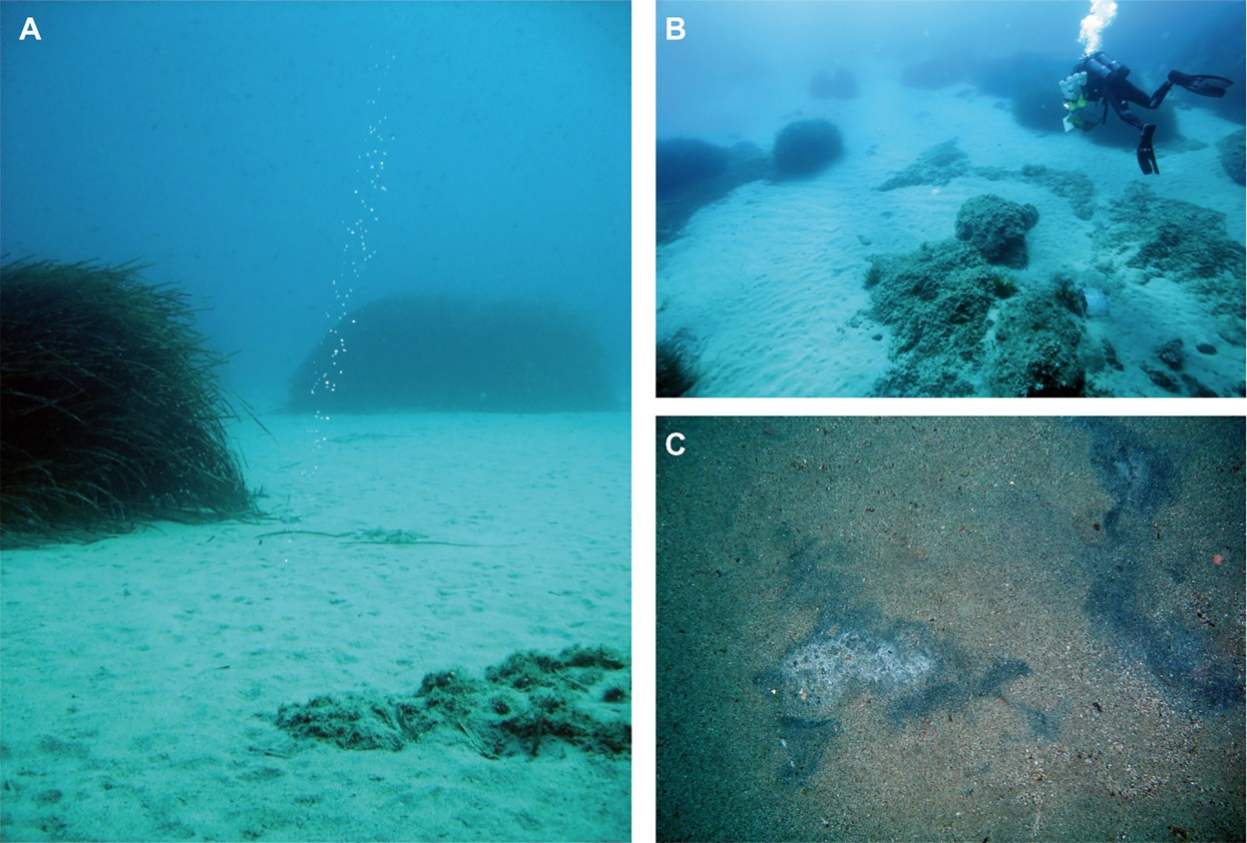
Underwater photographs. (A) Photograph showing seepage of CH_4_ bubbles. Seagrass on the left is ca. 1 m in height. (B) Overview of emission spot 2 and 3 and the distribution of sandy areas, dead seagrass meadow and living seagrass. (C) Black spots with white areas appearing during stable summer weather. White areas are a loose biofilm of *Beggiatoa*-like bacteria (the white area in the left centre is approximately 10 cm wide).

The number of emission spots in the studied CH_4_ seep area offshore Pomonte remained constant, and their location varied within half a square meter, since the beginning of the investigation in 2008. Within the 100 m^2^ study site we found seven bubble emission spots and 15 m southwest another three (Fig 2A and 2B). Bubble streams were found penetrating through the sediment and also through an old dead seagrass meadow (Fig 3A and 3B). The water depth of the seep site is 10–13 m, and the seafloor is thus affected by wave action. Water-column and porewater temperatures vary between 13 and 25°C during winter and summer, respectively. During summer months laminar current and fewer storms lead to more stable redox conditions just below the sediment surface, and dark-coloured spots and occasional white and olive-coloured biofilms are observed (Fig 3C). Patches of the seagrass *Posidonia oceanica*, as well as the remainders of dead and degrading seagrass meadow are characteristic for the area (Fig 3B).

At Pianosa, gas emission occurs at 10 to 45 m water depth out of carbonate sand and rocky outcrops. No samples have been retrieved from Pianosa so far but samples were taken from the Scoglio d’Africa site, where at 10 m water depth we observed frequent gas bubble emission from sandy sediments. Fishermen reported an outburst of gas at Scoglio d’Africa in March 2017, which was reported in local newspapers, but no quantifications of emission rates are presently available. All three sites of hydrocarbon seepage follow a North-South transect (Fig 1A).

### Sediment composition

The sediment at the Pomonte seep area consists of approx. 70% quartz, 20% feldspar, and 10% sheet silicates in addition to variable amounts of carbonate minerals described below. The sand has a permeability of 50.7 Darcy (= 5·10^−11^ m^2^) with no significant difference between emission spots and reference sites. Porosity is around 43% from 0 cm down to 60 cm in unaffected sediment, while it varies by ±10% at the emission spots. The sediment is sand with a median grain size of 430 μm at reference sites and 449 μm at emission spots. The grain size distribution is constant to 60 cm sediment depth.

**Fig 4:**
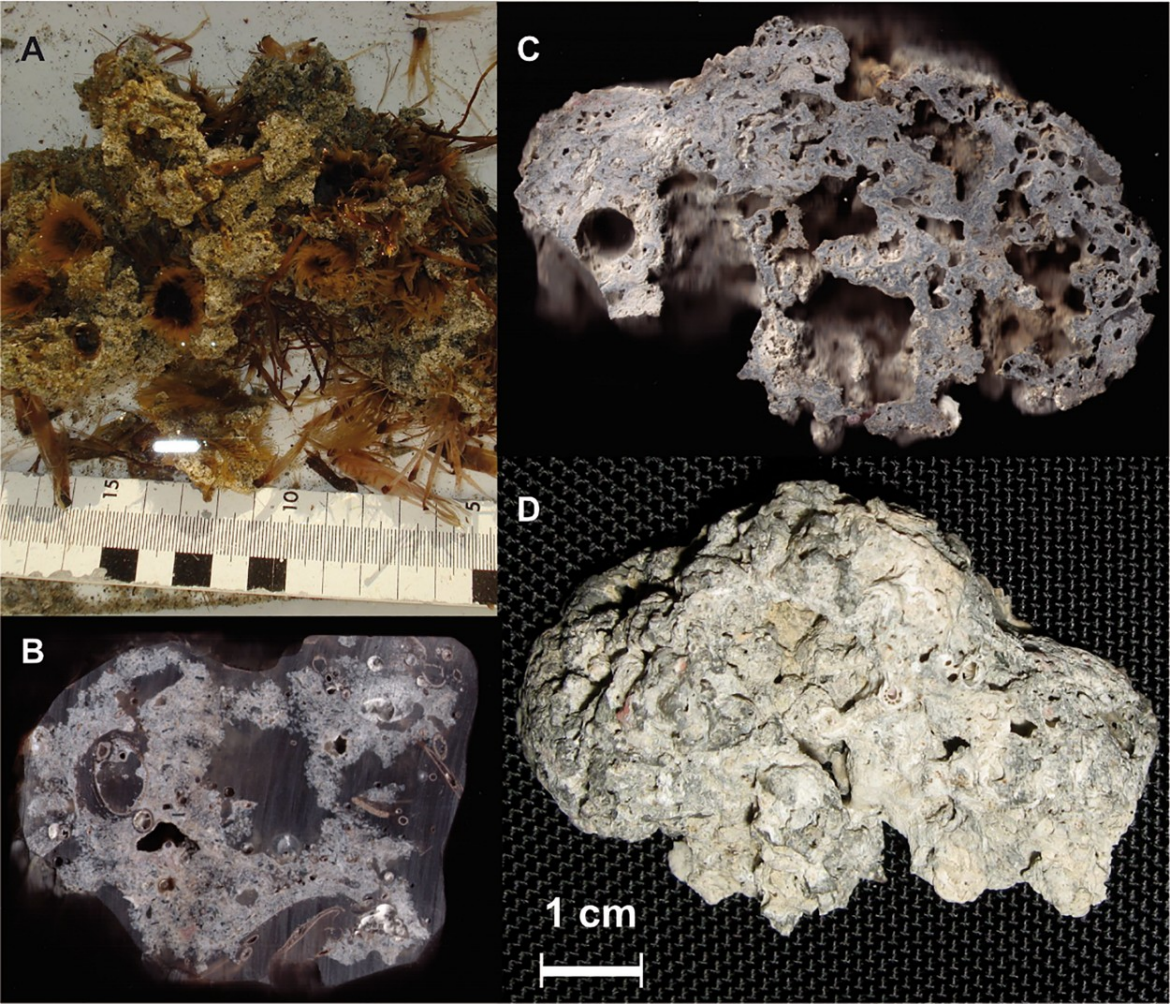
Photographs of the sediment at methane seeps. (A, B) Photograph of a clump of seagrass rhizomes cemented by aragonite from 10 cm sediment depth. (C, D) Hard cemented crusts containing debris of algae, calcified serpulid tubes. The entire structure is heavily encrusted by bryozoan colonies. The scale bar is valid for panels (C, D).

**Fig 5:**
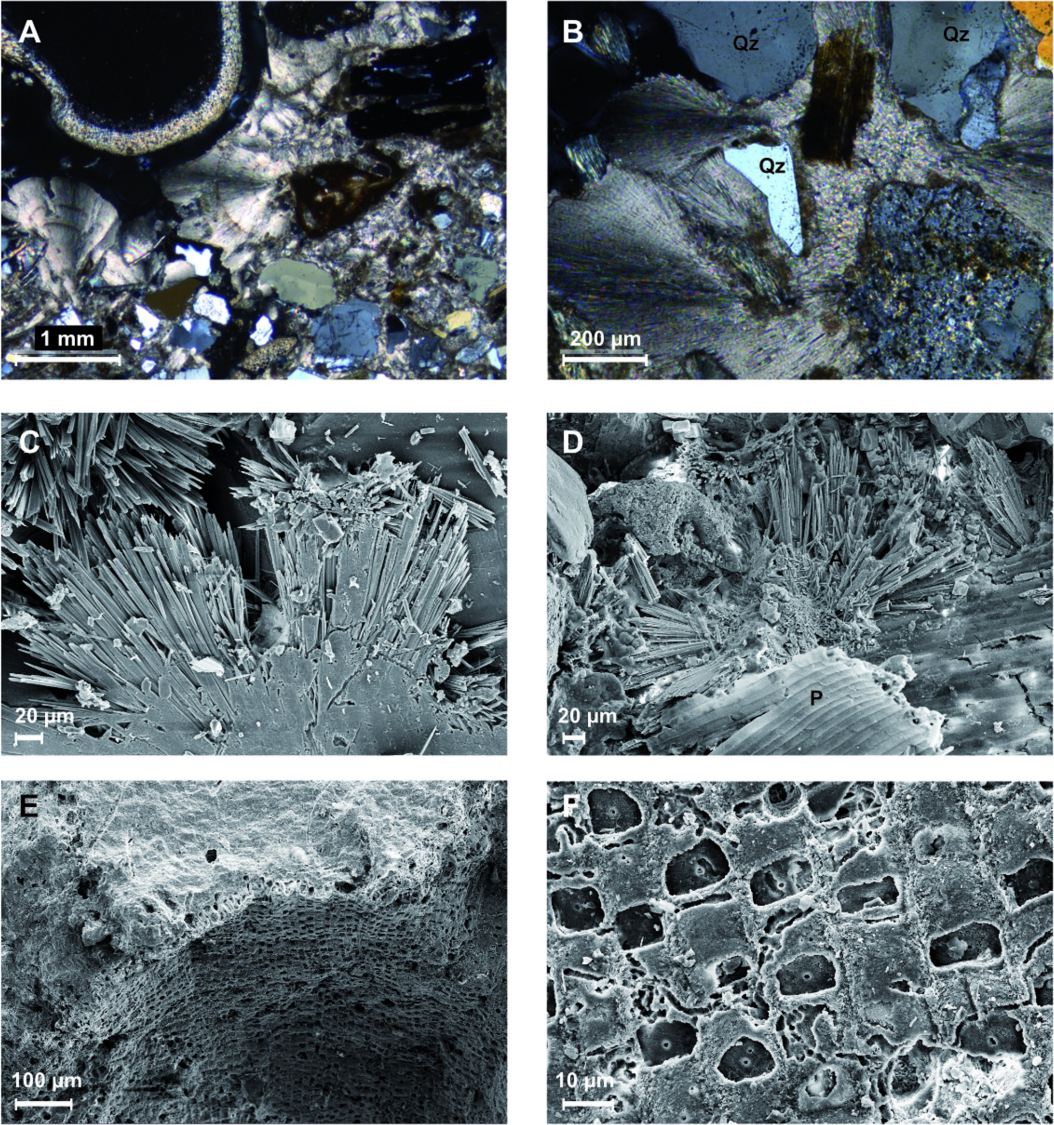
Microscope images of cemented sediment. (A, B) Thin section photomicrographs of the carbonate crust shown in Fig 4B. The fibrous aragonite crystal fans show a sweeping extinction under cross-polarized light. Several quartz grains (Qz) are cemented by the aragonite. In (A), the upper left quadrant shows a cross section through a calcified serpulid tube. (C, D) Scanning electron micrographs showing fibrous aragonite crystal fans. In (D), a fragment of seagrass (P) occurs within the aragonite cement. (E, F) SEM images of bryozoan colonies from the cemented crust in Fig 4C and 4D.

Carbonate-cemented sands were found within 10 cm of the gas emission spots (Fig 4). Cemented sand layers occur between 20 and 40 cm depth at emission spots 1, 2 and 3. Pieces are up to 10–50 cm^3^ and incorporate sand grains and seagrass rhizomes (Fig 4A and 4B). Other carbonate aggregates are encrusted with serpulid tubes and bryozoan colonies (Fig 4C and 4D). Thin section analysis (Fig 5A and 5B) and scanning electron microscopy (Fig 5C and 5D) revealed spherulitic cements with needle shaped microstructure. Some of the cemented sediments contain bryozoans (Fig 5E and 5F) and others seagrass rhizome fibres (Fig 5D). The carbonate consists of aragonite and high-magnesium calcite with 12–14% Mg; the latter mainly occurs in specimens with abundant bryozoans. In some pieces, framboidal pyrite (5 μm diameter) was found.

**Fig 6:**
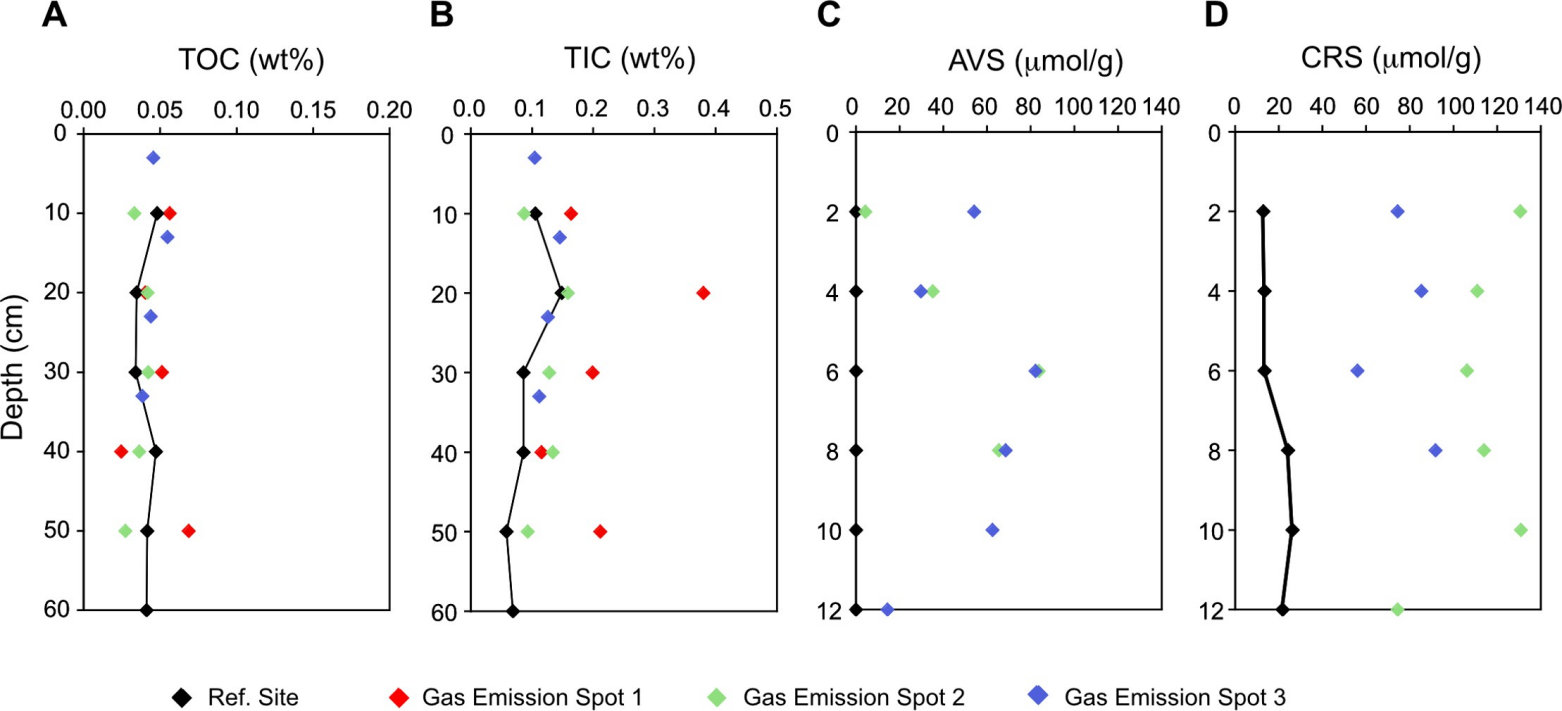
Sediment chemistry of bulk sediments from gas emission and reference spots. (A) Total inorganic carbon, (B) total organic carbon, (C) acid volatile sulphide and (D) chromium-reducible sulphur.

The TOC content of the sediments is less than 0.1 wt% (Table 1; Fig 6A). The mean TIC content is also below 0.1 wt%, but commonly reaches 0.2 wt% at the emission spots with a maximum of 0.4 wt% in TIC at emission spot 1 (Table 1; Fig 6B). At the emission spots AVS and CRS (Table 2; Fig 6C and 6D) reach concentrations of up to 60 and 130 μmol per gram sediment, whereas AVS is absent and CRS below 20 μmol g^-1^ at the reference spots.

**Table 1:** Total carbon (TC), total organic carbon (TOC), total inorganic carbon (TIC), and δ^13^C of the TIC at three gas emission spots and one reference spot (indicated in Fig 1). “Rep.” indicates measurements of separate samples from the same site.

Depth	TC	TIC	TOC	δ^13^C_TIC_	δ^13^C_TIC_ (Rep.)	δ^13^C_TIC_
(cm)	(wt%)	(wt%)	(wt%)	(‰ VPDB)		Selected fragm.
***Reference site 1***						
10	0.15	0.11	0.05			
20	0.18	0.15	0.03	-2.69		
30	0.12	0.09	0.03	-4.30		
40	0.13	0.09	0.05	-6.44		
50	0.10	0.06	0.04	-6.52		
60	0.11	0.07	0.04			
***Reference site 2***						
3				-6.87		
13				-4.72		
23				-4.41		
***Reference site 3***						
20				-4.12		
30				-6.17		
40				-5.72		
50				-5.43		
***Gas emission spot 1***						
10	0.22	0.16	0.06	-6.32	-15.50	-17.12
20	0.42	0.38	0.04	-7.36	-15.32	-16.63
30	0.25	0.20	0.05	-5.67		-15.27
40	0.14	0.12	0.02			-17.06
50	0.28	0.21	0.07			
60				-7.48		
***Gas emission spot 2***						
0	0.15	0.10	0.05			
10	0.20	0.15	0.05	-7.16	-14.67	
15						
20	0.17	0.13	0.04	-4.56	-14.33	
25						
30	0.15	0.11	0.04			
60				-9.70		
***Gas emission spot 3***						
10	0.12	0.09	0.03	-6.25		
20	0.20	0.16	0.04			
30	0.17	0.13	0.04	-6.23		
40	0.17	0.13	0.04			
50	0.12	0.09	0.03			

**Table 2:** Acid-volatile sulphide (AVS) and chromium-reducible sulphur (CRS) concentrations in bulk anoxic sediment at two gas emission spots and one reference spot (indicated in Fig 1).

Depth	AVS	CRS
(cm)	(μmol/g)	(μmol/g)
***Reference site 1***		
2	0.0	13.0
4	0.0	13.5
6	0.0	13.6
8	0.0	24.3
10	0.0	26.3
12	0.0	21.7
***Gas emission spot 2***		
2	4.3	74.4
4	35.2	85.3
6	83.7	56.1
8	65.5	91.7
***Gas emission spot 3***		
2	54.1	130.7
4	29.7	110.9
6	82.3	106.2
8	68.5	114.0
10	62.5	130.8
12	14.5	74.4

**Fig 7:**
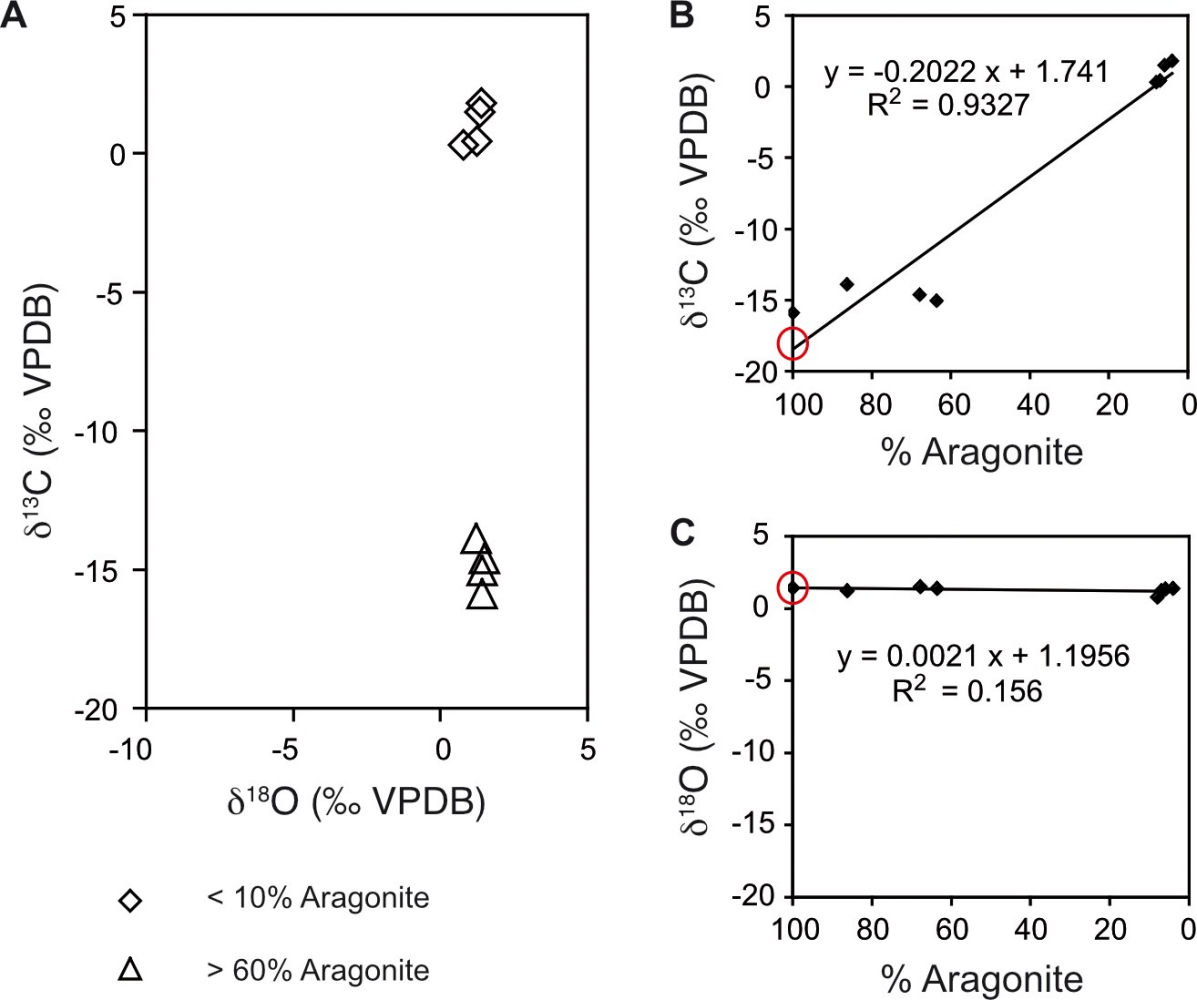
Isotope compositions of seep carbonates. (A) Cross plot of δ^13^C and δ^18^O from carbonates micro-drilled from several different cemented fragments. (B) The same δ^13^C values as in (A) plotted against the percentage of aragonite relative to all carbonate phases. Different aragonite contents are due to variable contribution of biogenic high-Mg calcite (probably from bryozoans). The isotopic composition of the pure aragonite cement can be extrapolated with the regression line. (C) The same for δ^18^O values. The regression line shows practically no slope between samples rich in Mg-calcite and samples rich in aragonite.

**Fig 8:**
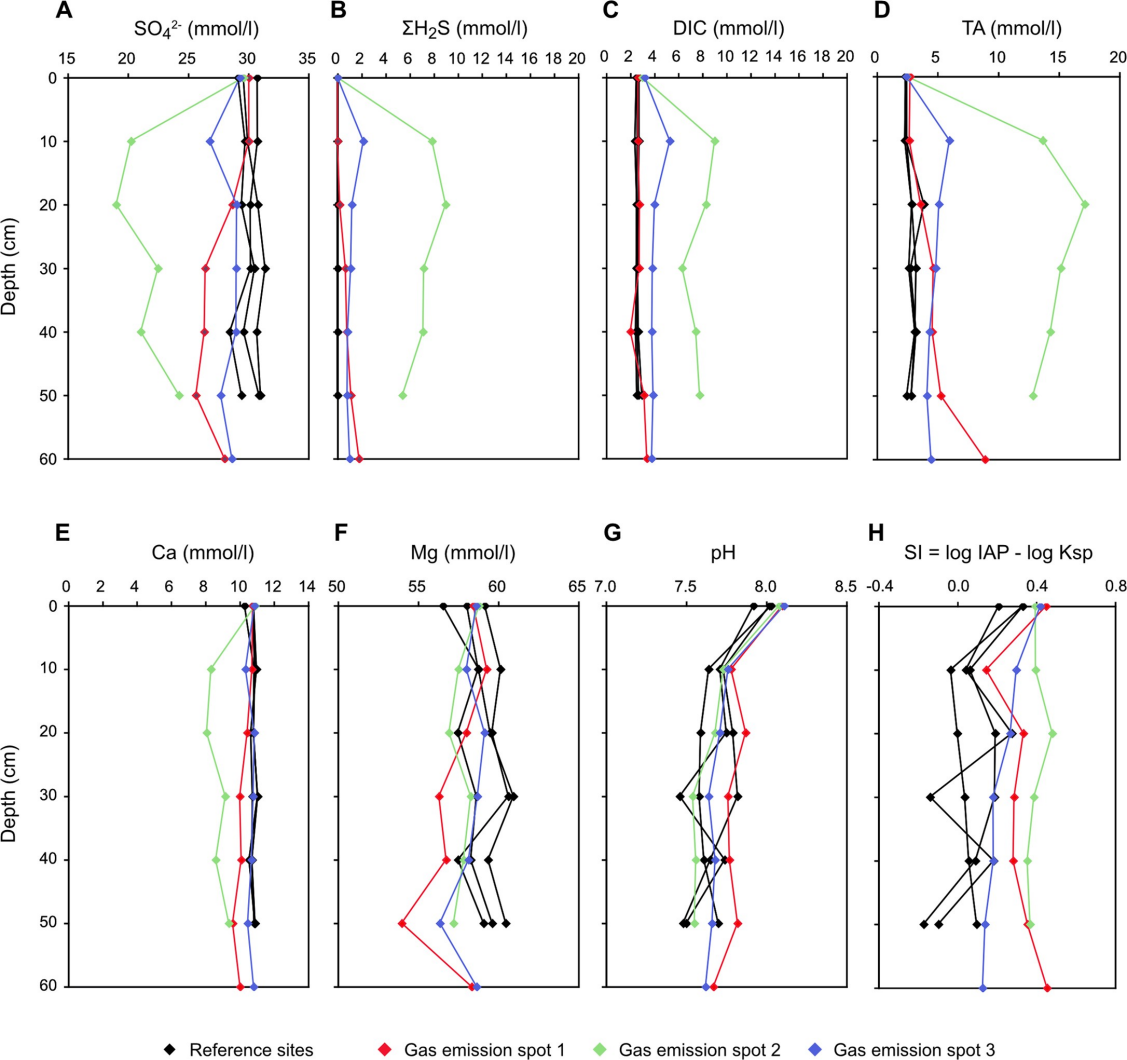
Porewater profiles at emission spots and control sites. Concentrations of SO_4_^2-^ (A), ∑H_2_S (B), DIC (C), TA (D), Ca (E), Mg (F), and pH (G) of porewaters sampled in at gas emission and reference spots 1–3 sampled in 2011. The saturation index with respect to aragonite (H) was calculated based on the measured pH and TA.

The carbonate crusts show a large scatter in δ^13^C values between -17 and +2‰ VPDB, while the δ^18^O values are uniform near 1.5‰ VPDB (Table 3; and cross plot in Fig 7A). Carbon isotope values reflect the relative contributions of authigenic aragonite and high-Mg calcite, with the latter being mainly derived from bryozoans (Fig 7B). The isotopic composition of the pure aragonite is in the range of -20 to -15‰. In contrast, the carbonate δ^18^O values do not show any relation to carbonate mineralogy (Fig 7C). δ^13^C determined from trace amounts of carbonate from the bulk sediment core samples from the emission spots shows less negative values that are similar to the composition in DIC (see below). The most negative DIC values are between -5 and -10 ‰ at 10 and 20 cm depth (Fig 8C).

**Table 3:** δ^13^C and δ^18^O of micro-drilled carbonates from crusts recovered from 64 cm depth at emission spot 2.

sample id	δ^13^C	δ^18^O
	(‰ VPDB)	(‰ VPDB)
***Micro-drilled samples***		
*Emission spot 2*, *64 cm sediment depth*		
Calcite rhizome	-14.61	1.49
Calcite nodule	1.50	1.34
PM 1	-15.04	1.40
PM 2	-15.88	1.41
PM 3	-13.89	1.22
PM 4	0.31	0.79
PM 5	1.81	1.40
PM 6	0.44	1.23

### Fluid chemistry

Porewater chemistry data are presented in S1 and S2 Tables and Fig 8. Sulphate concentration in Tyrrhenian seawater is about 29 mmol/l and is constant throughout the porewater profiles at the reference sites. At the emission spots sulphate is lowered to 20 mmol/l at 20 cm depth (emission spot 2, Fig 8A). While sulphide concentrations are below the detection limit at the reference sites, emission spot profiles show concentrations of up to 2 mmol/l at emission spots 1 and 3 and up to 8 mmol/l between 15 and 25 cm at emission spot 2 (Fig 8B). Dissolved CH_4_ concentrations reach up to 0.1 mmol/l at emission spot 1 and up to 0.3 mmol/l at emission spot 3, but only up to 0.14 μmol/l at control site 1 (S1 Table). DIC profiles show maximum concentrations between 5 and 9 mmol/l at emission spot 2 (Fig 8C). The sediment porewater profiles show DIC concentrations of around 2.5 mmol/l, similar to seawater above the emission spots. Total alkalinity is highest between 15 and 25 cm (Fig 8D) with a maximum of 17 mmol/l reached at 20 cm depth at emission spot 2. This TA surplus approximately matches the sum of sulphide and DIC, which are the main contributors to TA in marine porewater. The highest TA at the reference spots was less than 4 mmol/l. Dissolved Ca is depleted by 2 mmol/l at emission spot 2 below 20 cm, while other emission spots, as well as the reference spots, did not reveal concentration changes with depth (Fig 8E). A similar depletion as with Ca was also observed in the Sr profile (S1 Table). Also, Mg appeared to be slightly depleted at the emission spots, as opposed to the reference spots, but this depletion was not at the same depth as for Ca (Fig 8F). A pH value of 8.1 was measured in the water column (Fig 8G). In the porewater the pH values consistently decreased with depth to around 7.7 at all spots. Calculated SI values are between 0 and 0.5 for aragonite (Fig 8H) and calcite, and between 1 and 2 for dolomite (not shown) in seawater. In the porewater, SI decrease downward at the control spots with aragonite just around saturation and around 1 for dolomite. The saturation indices at the emission spots remain close to seawater level. At some depths at the emission spots SI values even exceed seawater levels.

**Fig 9:**
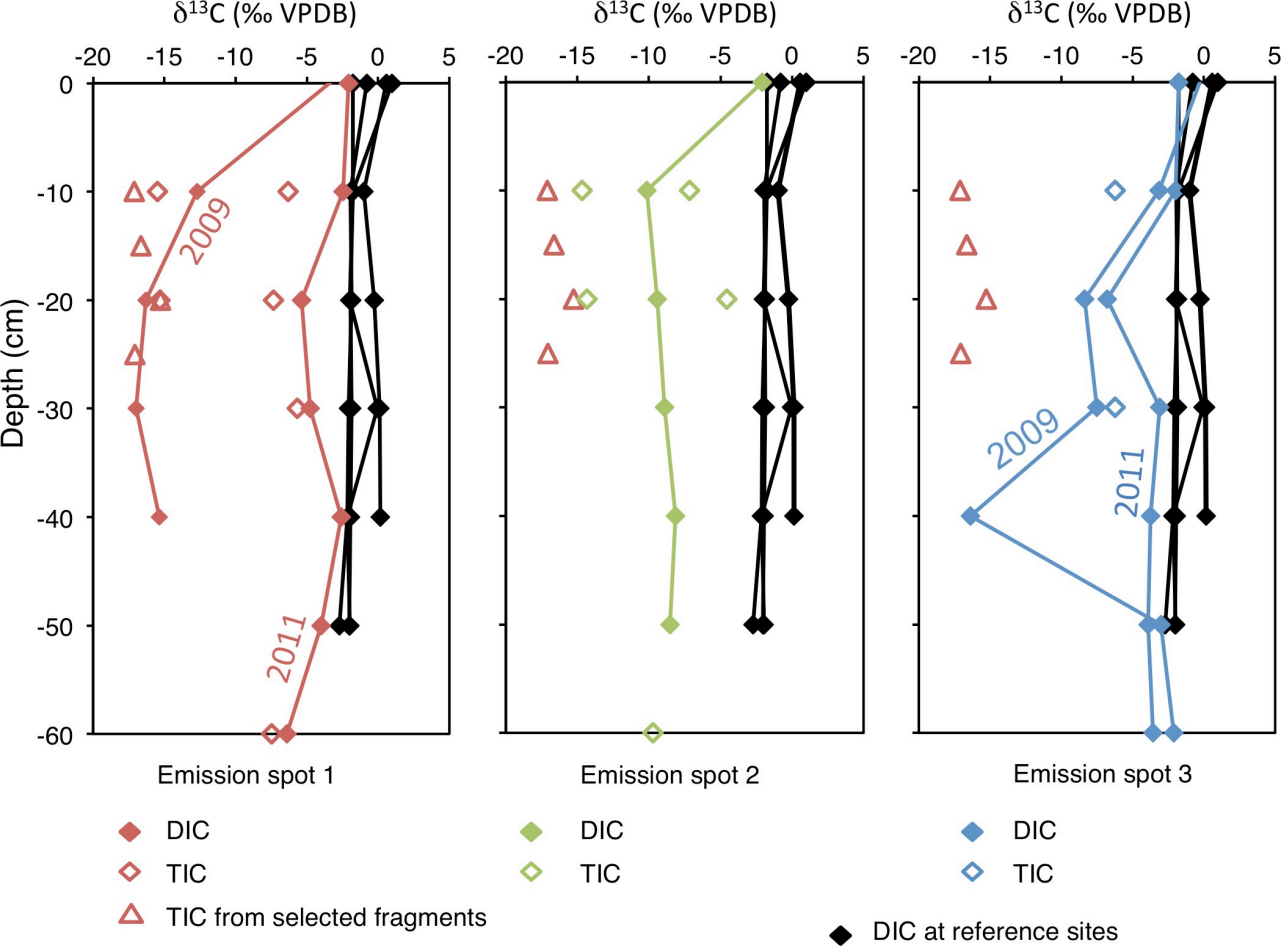
Isotopic compositions of dissolved inorganic carbon and carbonate at emission spots. δ^13^C values are plotted vs. depth for emission spots 1, 2, 3 and reference sites. The data are from porewater and sediment sampled in 2011.

**Fig 10:**
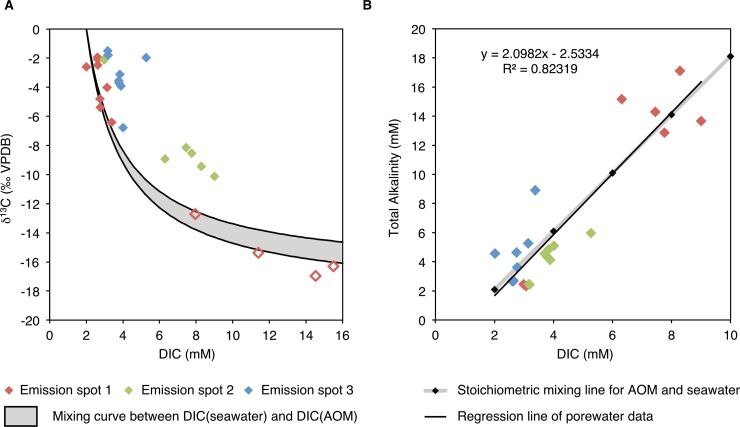
Carbon isotope compositions and inorganic carbon content of porewater at emission spots. (A) Cross plot of δ^13^C_DIC_ vs. DIC. Open symbols indicate samples taken in 2009. (B) TA vs. DIC. The regression line of all measurements shows almost a perfect match to the stoichiometric composition expected from the AOM reaction.

The δ^13^C composition of DIC is -3 to -7 ‰ VPDB at emission spots 1 and 3, and -10‰ VPDB at emission spot 2 (S1 and S2 Tables; Fig 9). Even more negative values occurred during a repeated sampling in 2011 with -15 ‰ at emission spot 1 (S1 and S2 Tables). Such negative values also occur in some of the carbonate cements micro-drilled from carbonate crusts (see above). At the reference spots, the values were -2‰ VPDB, which correspond to the value of modern seawater. δ^18^O_H2O_ values of all spots were between 1 and 1.5‰ (Vienna Standard Mean Ocean Water; VSMOW), which is the composition of Tyrrhenian Sea water (S1 Table). A correlation between δ^13^C and DIC, following a mixing hyperbola (Fig 10A), and a linear correlation between TA and DIC (Fig 10B) are observed.

**Fig 11:**
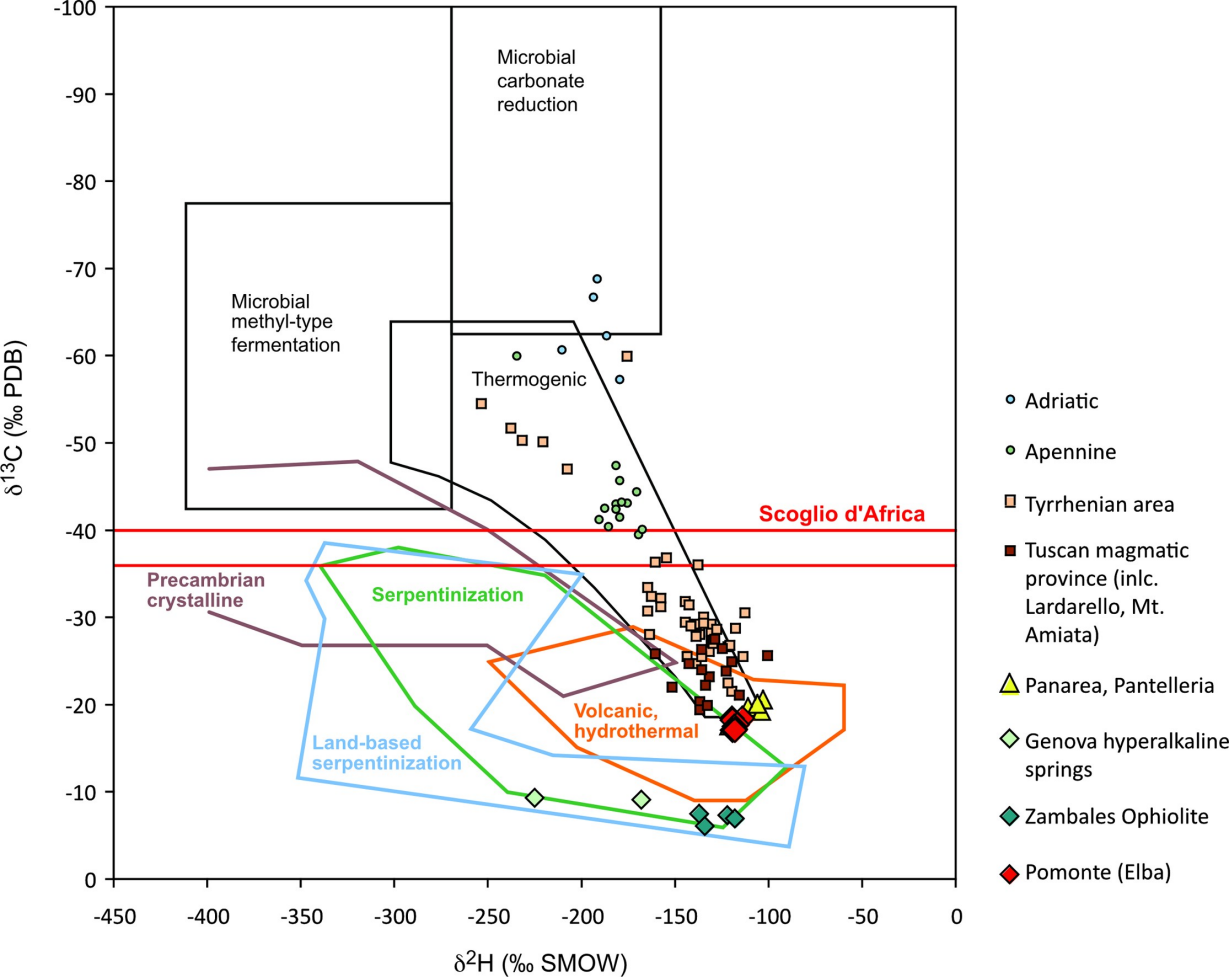
Schoell plot showing δ^13^C and δ^2^H compositions of seepage gas. δ^13^C and δ^2^H compositions of CH_4_ from the Pomonte seep and several sites from magmatic and thermogenic CH_4_ from the Tuscan, Tyrrhenian, Ligurian and Appenine regions compiled from [61]Gherardi et al. (2005), [60]Tassi et al. (2012), [78]Abrajano et al. (1988), and [41]Boschetti et al. (2013). The fields of microbial and thermogenic CH_4_ are from [28]Bradley and Summons (2010). Crystalline, serpentinization-derived, and volcanic/ hydrothermal CH_4_ fields are updated from [30]D’Alessandro et al. (2018).

The gas emission rate of the seven recorded gas emission spots was 234 ml m^-2^ d^-1^. The gas contains 65–73% v/v CH_4_ and 0.33 · 10^−3^% v/v ethane (Table 4). The gas also contains CO_2_, propane and other hydrocarbon gases, but concentrations were not measured. However, CO_2_-concentrations estimated from the mass 44 peak from stable isotope analysis are two orders of magnitude lower than CH_4_ concentrations. Stable carbon isotope analyses show values of around -17 to -18‰ for CH_4_ and around -22 to -24‰ for ethane (Table 4). Additional measurements of CH_4_ from emission spot 1 show rather constant δ^13^C values around -18 ‰ and δ^2^H values around -118‰ (Fig 11). Also CO_2_ gas shows negative values similar to CH_4_ (between -14 and -20‰). At the Scoglio d’Africa site the gas is composed of 75% v/v CH_4_ and 0.27 · 10^−3^% v/v ethane. This CH_4_ had δ^13^C values of -36 to -40‰, whereas the δ^13^C of ethane was slightly less negative (between -20 and -22‰). In contrast to the Pomonte seep site, the Scoglio d’Africa seep site showed CO_2_ with positive δ^13^C values of 16 to 22‰.

**Table 4:** (A) Gas concentration of methane and ethane as well as the δ^13^C-values of methane, ethane and carbon dioxide from the seep sites Pomonte and Scoglio d’Africa. (B) δ^13^C and δ^2^H of methane at Pomonte gas emission spots (locations indicated in Fig 1).

Site	CH_4_		C_2_H_6_		δ^13^CH_4_		δ^2^H_CH4_		δ^13^C_2_H_6_		δ^13^CO_2_		Comments
	(mmol/l gas)		(μmol/l gas)		(‰ VPDB)		(‰ VSMOW)		(‰ VPDB)		(‰ VPDB)		
***Pomonte***													
Seep 1					-18.4	±0.51	-113.9	±5					Imprint Analytics
(Replicates)					-18.4	±0.51	-119.5	±5					Imprint Analytics
					-18.2	±0.51	-119.2	±5					Imprint Analytics
					-17.7	±0.51	-116.7	±5					Imprint Analytics
					-17.5	±0.51	-118.3	±5					Imprint Analytics
					-17.2	±0.51	-117.7	±5					Imprint Analytics
					-17.1	±0.51	-119.2	±5					Imprint Analytics
					-17.1	±0.51	-118.0	±5					Imprint Analytics
Seep 1	28.46	± 1.61	14.30	± 0.02	-18.35	± 0.49			-24.05	± 0.38	-13.94	± 0.97	MARUM
Seep 2	25.73	± 1.66	13.15	± 0.85	-16.73	± 0.004			-22.38	± 0.06	-19.67	± 1.83	MARUM
Seep 3	25.82	± 5.24	18.76	± 2.66	-17.08	± 0.61			-24.27	± 0.14	-17.62	± 3.69	MARUM
***Scoglio d'Africa***													
Seep 1	30.72	± 3.30	13.52	± 0.14	-39.92	± 0.27			-21.57	± 0.09	16.37	± 0.21	MARUM
Seep 2	31.30	± 4.80	10.56	± 1.03	-35.51	± 0.35			-20.78	± 0.08	22.39	± 0.07	MARUM

### Potential sulphate reduction and methane oxidation rates in seep-related sediments

*In vitro* sediment incubation experiments show high AOM rates at emission spot 1 with a maximum of 39 μmol/l d in the porewater at 40 cm depth (Table 5). The recorded AOM rates are similar if measured via ^35^S and ^14^C tracer. In contrast, rates of organoclastic sulphate reduction measured without addition of CH_4_ are very low (max. 2.5 ±3.8 μmol/l d). No activity was detected at the control sites, even with CH_4_ added. Furthermore, supernatant of the active cultures showed active growth when inoculated into fresh medium.

**Table 5:** Rates of microbial turnover at gas emission spot 1 at 20, 40 and 60 cm below the sediment-water interface. Anaerobic methane oxidation rates were measured directly via ^14^C-labelled methane. Sulphate reduction rates measured by radiolabelled ^35^S-tracer indicate the overall rate of sulphate reduction, whereas the SRR in incubations without CH_4_ added indicates the rate of organoclastic sulphate reduction. No other substrate than CH_4_ was added.

Depth	Meth-ox		R_SR_ with CH_4_		R_SR_, no CH_4_	
(cm)	(nmol/g d)		(nmol/g d)		(nmol/g d)	
***Gas emission spot 1***						
20–25	1.6	±0.9	9.0	±3.5	7.0	±3.3
40–45	144.9	±24.5	198.3	±45.7	13.0	±19.6
60–65	136.4	±12.7	69.7	±20.7	12.6	±1.8
***Rates of turnover per volume of porewater***						
	(μmol/l d)		(μmol/l d)		(μmol/l d)	
20–25	0.3		1.8		1.4	
40–45	28.3		38.8		2.5	
60–65	26.7		13.6		2.5	

## Discussion

### Origin of the methane

#### Abiotic methane seepage at Pomonte

Methane seepage occurs about 200 m off the coast of Pomonte, in an area of ca. 1000 m^2^ and at a distance of approximately 20 metres from the next bedrock outcrop. From the south-eastern shelf it is known that the sedimentary cover is only up to 20 m thick at 100 m water depth ([79]Akal, 1970), and [80]Badran et al. (2008) report a sediment thickness of 7.5 m at the same water depth. Even though the Pomonte site is in shallow water and sunlight supports microphytobenthos, we exclude photoautotrophic biomass as a source of sufficient TOC to drive methanogenesis, as it has been shown that rates of photosynthesis are low and nutrient-limited ([81]Sevilgen, 2008). In addition, the siliciclastic sands contain extremely low amounts of TOC (<0.06 wt%). Given the geological setting and the isotopic composition of the CH_4_, it is highly unlikely that it originates from methanogenesis within the thin sediment cover at this site. We also exclude the possibility that degradation of seagrass could be the source of CH_4_, as its rhizomes and roots are known to degrade very slowly ([82]Pergent et al., 1994; [83][84]Mateo et al., 1997, 2006), and because we did not detect seagrass debris in the sampled sediment. Furthermore, all sites, where buried degrading seagrass was found, are not depleted in sulphate, which would be a prerequisite for methanogenesis. From that we exclude formation of CH_4_ anywhere near the sediment surface or the sediment overlying the bedrock. Instead CH_4_ must originate from a deeper source that must be within the bedrock.

The underlying bedrock on the southwestern side of Elba consists of an ophiolite sequence with a contact metamorphic zone adjacent to the Monte Capanne pluton. Although the nature of the bedrock directly underlying the seep area is not known, we can assume that the bedrock is low in organic carbon. Of the ophiolitic units, the only sedimentary rock type known to contain sufficient organic matter in the region are the Argille a Palombini, but it occurs only outside of the displayed area (Fig 1B). Also, the Palombini shale is highly metamorphosed in the direct vicinity of the Pomonte site, which makes it an unlikely source of organic matter.

The abundance of methane relative to that of the next heavier alkanes can be used to distinguish between different sources. The gas sampled at Pomonte shows a high CH_4_/(C_2_H_6_+C_3_H_8_) ratio with nearly 2000-times more CH_4_ than ethane and butane, providing further evidence that it is not thermogenic. Thermogenic gas typically shows a low CH_4_/(C_2_H_6_+C_3_H_8_) ratio (<100) (e.g. [85]Whiticar and Suess, 1990). We therefore exclude the possibility that the seep methane is derived from thermal degradation of organic carbon in sediments at depth at the Pomonte site.

For the same reasons, it can be excluded that the seepage is due to exsolution of CH_4_ from gas hydrate. Furthermore, a microbial source of the seep gas is highly unlikely, as microbial hydrocarbons show CH_4_/(C_2_H_6_+C_3_H_8_) ratios that are much higher than what we observed.

Further information on the origin of the CH_4_ may come from its isotopic composition: The CH_4_ at Pomonte shows a carbon isotope composition (δ^13^C ≈ -18‰) that is much less negative than typical CH_4_ at sediment-derived hydrocarbon seeps. Although high δ^13^C_CH4_-values (and probably also high δ^2^H_CH4_-values) may result from kinetic fractionation during AOM (e.g. [11]Whiticar, 1999), inorganic carbon in the porewater and the carbonates at the Pomonte seep show minimal fractionation with respect to the CH_4_ (cf. Table 4 and electronic supplement). This is because most likely AOM from a stream of gas bubbles is controlled by the amount of CH_4_ that gets dissolved in the porewater. AOM in the sediment is then limited by the rate of dissolution, and CH_4_ undergoes a complete Rayleigh fractionation within the very small dissolved CH_4_ pool, resulting in a zero isotope effect. This is in agreement with the observation that often large amounts of CH_4_ by-pass AOM in the porous sediment ([86]Niemann et al., 2006; [87]Wegener, 2008) and the distance, which the CH_4_ escaping the bedrock passes through porous sediments, is minimal at this site.

As a thermogenic or microbial origin of the methane at Pomonte can be ruled out, it is likely that the gas seeps are due to abiotic processes in a fluid-rock system. The composition of the Pomonte seep gas and the isotopic composition of the CH_4_ clearly support the idea of an abiotic origin (cf. [17]Etiope and Sherwood-Lollar, 2013). In the updated Schoell-plot (Fig 11) δ^13^C_CH4_ vs. δ^2^H_CH4_ values plot at the lower margin of the abiogenic serpentinization-derived CH_4_ field, although the δ^13^C_CH4_ values are somewhat more negative than for some typical hyperalkaline ultramafic systems, such as the Zambales Ophiolite or hyperalkaline springs at Voltri. More diagnostic for abiotic gas is the inverse correlation of δ^13^C values with increasing carbon number of hydrocarbon gases as shown in Table 4 ([88]Des Marais et al., 1981; [89]Sherwood Lollar et al., 2002; [90]Pan et al., 2006; [31]McCollom and Seewald, 2007; [32]Konn et al., 2015). In summary, the geological setting, the gas composition and the isotope signatures are consistent with an abiotic origin of the CH_4_ at the Pomonte seep.

#### Conceptual model for abiotic methane generation

With the isotopic composition of the methane at Pomonte being between that of serpentinite-hosted gas seeps and the distal end of other hydrothermal sources (Fig 11), the abiotic processes that led to the methane formation are not clear. In principle, different mechanisms and, accordingly, different conceptual models may be suggested based the geological setting and the gas compositions, which require a magmatic, metamorphic, high-T hydrothermal or low-T hydrothermal origin. Different scenarios will be discussed in this section.

Abiotic methane may come from magmatic degassing or from metamorphic reactions. Magmatic degassing will commonly release a gas that has a low methane concentration and is rich in CO_2_ ([31]McCollom and Seewald, 2007; [91]Fiebig et al., 2015). Magmatic degassing will also be confined to areas of active magmatism. The heat flow in the study area is relatively low ([92]Della Vedova et al., 2001), so it is unlikely that the gas emissions at Pomonte are related to fumarolic activity due to magmatism. Both, the geological setting and the composition of the gas at Pomonte are hence inconsistent with a magmatic source of the methane.

**Fig 12:**
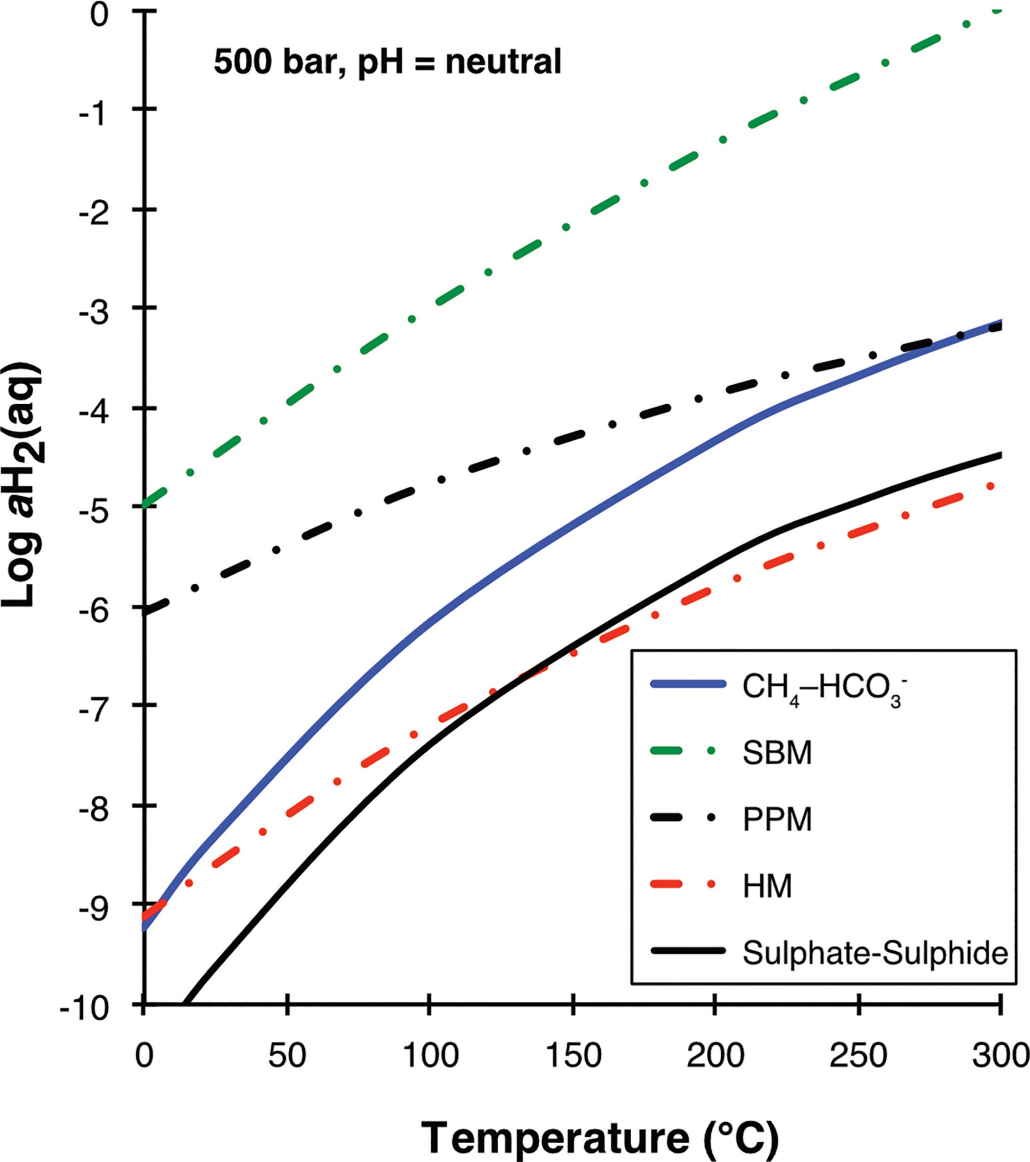
Activity of dissolved dihydrogen (H_2_(aq)) versus temperature calculated for different mineral buffers (dash-dot lines). HM: hematite-magnetite, PPM: pyrite-pyrrhotite-magnetite, SBM: serpentine-brucite-magnetite. Unit activities of the solids were assumed, except for SBM: a_Fe-Brc_ = X_Fe_ = 0.1, a_Fe-Srp_ = (X_Fe_)^3^ = 0.03^3^ = 0.000027. The mineral buffers represent the reduction potential of granitic (HM), gabbroic (PPM) and ultramafic (SBM) rocks. Also shown are the divides between the predominance fields of CH_4_ and HCO_3_^-^ (in blue) and SO_4_^2^- and H_2_S. Those lines were calculated for the reactions HCO_3_^-^ + 4 H_2_(aq) + H^+^ = CH_4_(aq) + 3 H_2_O and SO_4_^2-^ + 4 H_2_(aq) + 2 H^+^ = H_2_S(aq) + 4 H_2_O under the assumption that the activities of HCO_3_^-^ and CH_4_(aq) (or sulphate-sulphide) are on par and pH is neutral. The HCO_3_^-^ - CH_4_(aq) divide for fluids with a CH_4_/ HCO_3_^-^ ratio of 10,000 would be shifted upward from the plotted line by 1 unit in Log aH_2_(aq). The calculation results plotted suggest that gabbroic rocks support the production of hydrogen concentrations high enough to drive CO_2_ reduction to CH_4_ at temperatures <250°C. All calculations were conducted using SUPCRT92 [73](Johnson et al., 1992).

**Fig 13:**
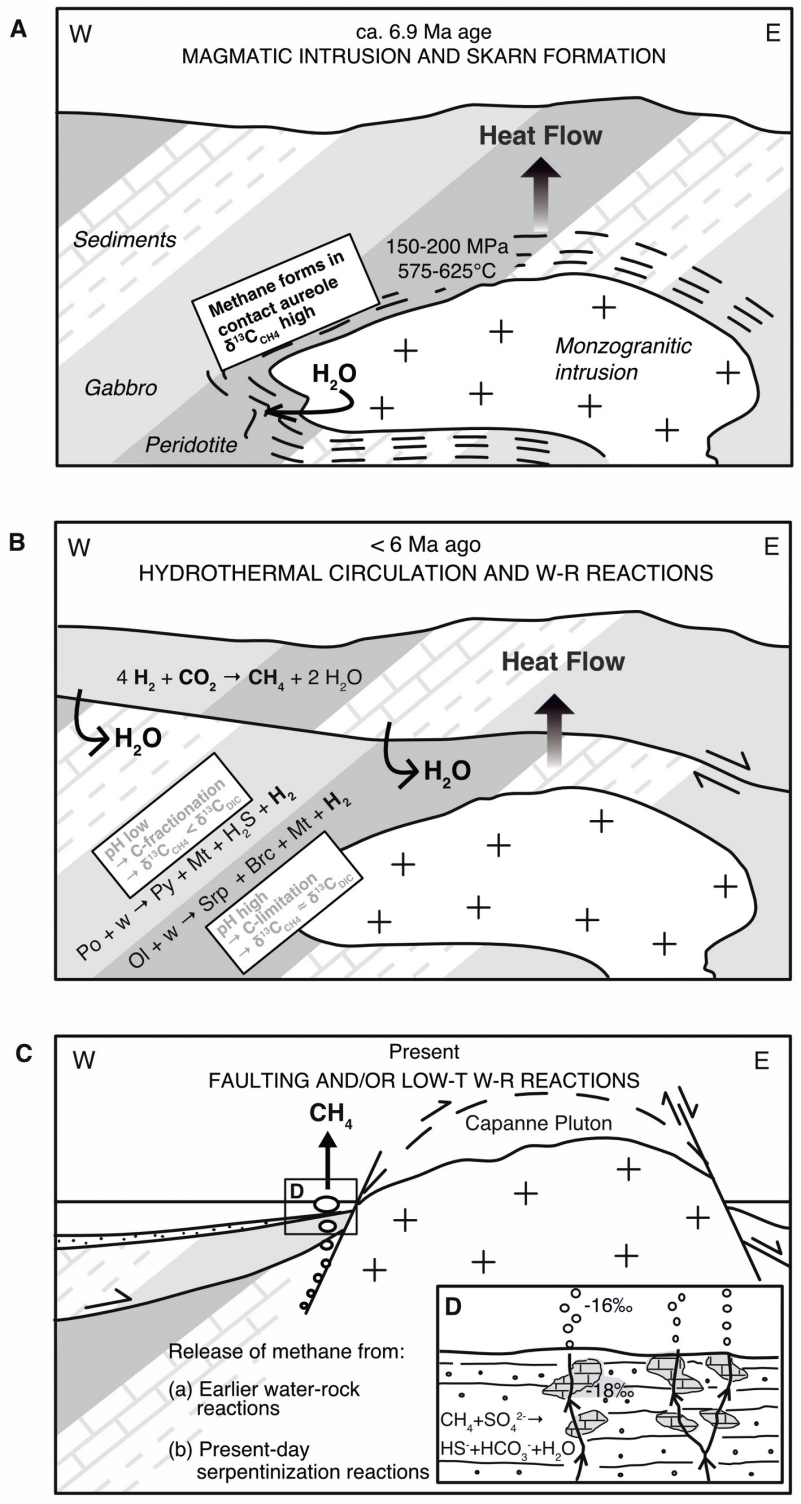
Conceptual models for abiogenic methane generation and seepage at Pomonte, Elba. (A) Intrusion of the Capanne pluton ca. 6.9 Ma ago. CH_4_ was generated in the contact metamorphic zone (dash-signature) due to reaction with rocks of the Ligurian ophiolite sequence, including peridotite, gabbro but also shale and carbonates. Small amounts of CH_4_ are still preserved in fluid inclusions. (B) A high heatflow in proximity to the intrusion caused the onset of a hydrothermal fluid flow along tectonic fractures during unroofing. Methane may have been generated by hydrothermal reactions with ultramafic rocks, and possibly with the whole suite of host rock once the temperature dropped below ca. 300°C. Reaction with different rock types at different temperatures affects the pH, CO_2_-availability and, thus, the δ^13^C_CH4_. (C) The present state where no elevated heatflow is recorded. Unmixing and degassing of CH_4_ may occur from deep hydrothermal reservoirs or by segregating from moderately alkaline fluid during low-temperature serpentinization. The inset (D) shows a close up of the seep area, with brick signature indicating the precipitation of authigenic aragonite cement. In absence of hyperalkaline fluid, carbonate precipitation is induced by AOM. Abbreviations: Brc = brucite Mt = magnetite, Ol = olivine, Po = pyrrhotite, Py = pyrite, Srp = serpentine, w = water. The geology is based on the tectonic model of [50]Smith et al. (2011) and is not to scale.

As far as metamorphic processes are concerned, there is a plethora of reactions that can produce methane. One critical parameter is the reducing capacity of a rock. As shown in Fig 12, mineral assemblages found in ultramafic rocks will support CO_2_ reduction to methane over a large range of temperatures, while those of mafic rocks do so only at low temperatures. Felsic rocks do not support methane generation under any circumstance. What this tells us is that reactions of water with certain lithologies of the ophiolitic unit exposed in the Pomonte area can produce methane, but reactions of water with the Monte Capanne pluton itself cannot. One possibility is that the CH_4_ was produced during high-temperature metamorphic reactions. [93]Lazar and Manning (2005) stated that, in crustal rocks, abiotic methanogenesis commonly requires high temperature processes. During the intrusion of the Monte Capanne pluton ca. 6.9 Ma ago ([55]Dini et al., 2002), rocks of the ophiolite sequence were exposed to 150–200 MPa and 575–625°C in the contact aureole of the intrusion ([94]Rossetti et al., 2007). In the village Pomonte, close to the seep site, various indications of contact metamorphosis are visible ([46]Frisch et al., 2008). [58]Rossetti and Tecce (2008) found methane in fluid inclusions in metasedimentary skarn, showing homogenization temperatures up to 580°C. These authors suggest that the methane formed from reduction of CO_2_ in the escaping magmatic fluid, and that the reducing power was generated from converting FeO in precursor minerals to Fe_2_O_3_ in metamorphic garnet. It is, hence, conceivable that the seep CH_4_ at Pomonte is derived from rocks in the contact aureole of the Monte Capanne pluton (Fig 13A). What would cause the release of CH_4_ observed today is not clear, though. The idea of seepage of fossil methane that formed during earlier metamorphic reactions is difficult to reconcile with the continuous seepage required to make the carbonate deposits at Pomonte. Repeated cracking of rock and opening of inclusion in which the methane is trapped would be required. To test the feasibility of this hypothesis one could look for microseismicity, which should be generated in association with such cracking events.

An alternative model for methane generation could be through hydrothermal reactions between water circulating at depth through hot basement during uplift and unroofing of the batholith (Fig 13B). In the aftermath of the Capanne intrusion, the heatflow was still high which could have caused a convection of water through the ophiolithic rocks of the Ligurian Nappes. However, in absence of the metamorphic reactions mentioned above, only a limited number of reactions are available that would develop sufficient reducing power to drive methanogenesis. While ultramafic rocks would be most efficient over the entire temperature range, mafic lithologies or even the Palombini shale would be possible candidates for abiotic methane formation (driven by the pyrrhotite-pyrite-magnetite buffer indicated in Fig 12) as the temperatures falls below 300°C during that stage of the geologic evolution of western Elba.

Clearly, the most efficient process to produce abiotic methane would be serpentinization of ultramafic rock. The calculation results presented in Fig 12 show that serpentinization reactions would buffer hydrogen activities of the interacting fluids to values well within the CH_4_ stability field down to relatively low temperatures. Such a scenario, as shown in Fig 13C, would be plausible, given that ultramafic rocks are present in the larger Pomonte seep area, and would be supported by the common occurrence of methane seepage in ultramafic units of many ophiolites around the globe. It is not entirely clear how exactly this methane may form, as recent studies suggest that kinetics of CH_4_ generation is exceedingly slow in homogeneous aqueous systems at low temperatures ([21]McCollom, 2016). As an analogue, in the <100°C hydrothermal vents at Lost-City, CH_4_ has apparently formed at higher temperatures, around 300°C, as shown by clumped isotope data ([95]Wang et al., 2018). But other clumped isotope data is consistent with a low-temperature origin of the methane ([17]Etiope and Sherwood-Lollar, 2013). The presence of a gas phase and mineral catalysts are likely required for abiotic methane formation to by-pass the kinetic bottleneck in the reactions of dissolved species.

The problem with the described scenario is, however, that no indications of a hyperalkaline fluid were detected. A system of intense serpentinization at relatively shallow depth that would produce the amounts of CH_4_ detected usually would produce highly alkaline fluids with a pH of 10–12, as observed in the Ligurian Ophiolites. But most serpentinite-hosted systems have gas escape as separate phase, so this is a feasible physical mechanism that could segregate methane-rich gas from interacting alkaline water in the place where serpentinization takes place. This would mean that there is no hydraulic head driving the upflow of water, like e.g., in the Bay of Prony serpentinization system ([37]Monnin et al., 2014), and that there is no thermally driven buoyant flow (like at Lost City) either. In absence of a heat flow anomaly the fluids in the basement would be stagnant and, hence, unable to seep like the buoyant gas phase does. This may be supported by the depletion of the seep gas in CO_2_, which is largely trapped as carbonate due to alkaline conditions at depth. The small amounts (ca. 1%) of CO_2_ detected in the bubbles may originate from partitioning of CO_2_(aq) in seawater into the CH_4_ bubbles.

A possible indication for the pH conditions during methanogenesis may come from the δ^13^C_CH4_ values of around -18‰, which is lower than the most positive values measured in abiotic methane and also lower than a presumed inorganic carbon source of ca. 0‰, both of magmatic or marine origin. Assuming that organically derived methane has no influence, as discussed above, ^13^C-depletion of CH_4_ is probably the result of a kinetic fractionation during methane generation. As the pool of DIC is limited due to hyperalkalinity caused by serpentinization, the cumulative isotope effect will diminish as a result of a Rayleigh effect. Hence, the δ^13^C of CH_4_ may serve as indicator for pH conditions and DIC limitation during abiotic methanogensis. Indeed, different mineral reactions have different effects on the pH. Mineral reactions in basaltic or pelitic rocks that depend e.g. on the pyrit-pyrrhotite-magnetite buffer shown in Fig 12 drive the pH to moderate values. In contrast, reactions with ultramafic rocks may develop extremely high pH-values, although, this depends on the composition. Harzburgite produces lower pH-values than lherzolite, and both types produce a moderate pH at elevated temperatures as brucite becomes instable ([96]Klein et al., 2013). Both rock types occur as part of the Ligurian ophiolite sequence at Elba, but also methanogenesis at elevated temperatures cannot be excluded.

While it is still not entirely clear which of the three models outlined here, and summarized in Fig 13, applies for the methane seeps observed at Pomonte, our preferred scenario, consistent with all observations mentioned above, would be a reaction of hydrothermal water with ultramafic rock producing alkaline conditions at depth, and segregation of CH_4_ gas under stagnant conditions. Methane generation may have occurred at earlier times under elevated heat flow or due to admixture of less hyperalkaline fluid from the alteration of harzburgitic, mafic or pelitic rocks at lower temperatures. A clearer picture awaits further investigation.

#### Origin of methane at Pianosa and Scoglio d’Africa

The observation of CH_4_ seepage at Pomonte raises the question whether this activity is part of a larger seep system. Therefore we briefly discuss observations and results from CH_4_ seeps at Pianosa and Scoglio d’Africa. Also for Pianosa and Scoglio d’Africa, showing a very thin recent sediment cover, a generation of CH_4_ in the surface sediment can be excluded. At Scoglio d’Africa the underlying bedrock consists of Triassic-Liassic limestone, at Pianosa of Cenozoic deposits, but it remains unclear whether an organic source is present in the bedrock at those sites.

The gas sampled at Scoglio d’Africa also shows a high CH_4_/(C_2_H_6_+C_3_H_8_) ratio with nearly 2000-times more CH_4_ than ethane and butane. Small amounts of CO_2_ (in the order of 1%) were detected during isotope measurements at both sites. According to the isotopic difference between CO_2_ and CH_4_ (Δ^13^C_CO2-CH4_) of ca. 60‰ at Scoglio d’Africa, an equilibrium temperature below 100°C can be calculated by the equation of [97]Horita (2001). The positive δ^13^C_CO2_-values at Scoglio d’Africa would be consistent with an origin from microbial methanogenesis. At least, microbial CH_4_ may contribute to the more negative δ^13^C_CH4_-values at Scoglio d’Africa (≈ -40‰), and the positive correlation of isotope values with hydrocarbon chain length also would support a microbial origin.

With the present data set available, only preliminary conclusions are possible. A complete gas analysis will be necessary to shed light on Scoglio d’Africa and Pianosa seep systems.

### Anaerobic methane oxidation at methane seeps

At the emission spots of the Pomonte seep, depletion of sulphate in the porewater, presence of dissolved sulphide, framboidal pyrite and elevated AVS and CRS contents clearly indicate ongoing sulphate reduction. Despite rather high permeability and flushing of the seafloor during winter storms, anoxic conditions prevail in less than 10 cm below the sediment surface. During summer time, more stagnant conditions lead to local anoxia at the sediment surface, visible as black areas (Fig 3C). The TOC content of the sediments is too low to provide sufficient substrate to drive significant CH_4_ production or organoclastic sulphate reduction. Typical vertically declining sulphate concentrations including sulphate methane transition zones were not observed. Instead we observed concentric sulphate depletions surrounding the focused CH_4_ emissions from the basement through conduits. This hypothesis is confirmed by high CH_4_-dependent sulphate reduction rates (39 μmol/l d) as determined in radiotracer experiments, whereas in incubations without CH_4_, rates were low (2.5 μmol/l d). At the reference site, both rates were low, showing that long CH_4_ exposure is required to establish AOM communities. This finding is consistent with a recent microbial ecology study ([44]Ruff et al., 2016) showing an average of 2.8 · 10^8^ microbial cells cm^-3^, including several representatives of the ANME group typically found in consortia of CH_4_-oxidizing archaea and sulphate-reducing bacteria in AOM zones. The *in vitro* activity of AOM in the sediment is also similar to rates measured at other seeps ([98]Knittel and Boetius, 2009; [86]Niemann et al., 2006; [99]Omoregie et al., 2008).

The stoichiometry of sulphate depletion and HS^-^, DIC and TA increases (Fig 8) is consistent with the AOM stoichiometry, producing two moles of TA per mole of DIC:

$${CH}_{4}+{SO}_{4}^{2-}\to {HCO}_{3}^{-}+{HS}^{-}+H_{2}O$$ (6)

It is the excess of TA over DIC that increases the saturation state with respect to carbonate minerals ([100]Meister, 2013). A linear correlation of the DIC vs. TA (regression line) with the ratio expected based on a 1:2 stoichiometric ratio due to the AOM- reaction is observed (Fig 10B). Due to very low TOC contents, other respiration mechanisms are irrelevant for DIC production, hence negative δ^13^C values of DIC (min. -17‰) result almost exclusively from oxidation of CH_4_. Fractionation between DIC and CH_4_ is not observed, probably because the reaction is dissolution-limited (see discussion above). Diffusive mixing occurs with DIC in seawater with a δ^13^C_DIC_ value around 0‰ VPDB but as the concentration of DIC in seawater is only around 2 mmol/l, its contribution to the pore fluid is minor. Ideally, the mixing results in hyperbolic porewater profiles, where negative δ^13^C_DIC_ values are reached at very shallow depth (Fig 10A). Such ^13^C-depleted DIC has accumulated in the porewater at emission spot 1 sampled in October, 2009, as opposed to porewater sampled in April 2011. Emission spot 3 shows the opposite trend, i.e. a decrease over time, which is reflecting the dynamic behaviour of the CH_4_ emission (Fig 9). CO_2_ gas shows similar isotope values as CH_4_ at the Pomonte seep, however, its concentration is two orders of magnitude lower than CH_4_ and its influence on δ^13^C_DIC_ is therefore negligible.

### Methane derived aragonite cements

Several crusts and fragments of partially cemented sand were recovered from the three investigated seep emission spots (Fig 5). Two different carbonates are observed: Bryozoan colonies and botryoidal cements consisting of fans of needle-shaped crystals. Point measurements by EDX confirm that the Mg is associated with the bryozoans. The aragonite detected by XRD can therefore by assigned to the crystal fans. Botryoidal aragonite occurs at particular depths within the emission spots or scattered in the surrounding of active seeps but they are not present at the reference spots. Extrapolated δ^13^C-values of pure endmember Mg-calcite are near to normal seawater values, whereas aragonite shows δ^13^C values around -18 ‰ VPDB, very close to the values measured in CH_4_ (Fig 7B). Taking into account an uncertainty of a few ‰ due to fractionation between carbonate and DIC, this suggests that the DIC from which the aragonite precipitated is almost entirely derived from CH_4_.

Based on the measured oxygen isotope composition of the porewater and aragonite, and assuming that aragonite precipitated in isotopic equilibrium with the porewater, the calculated carbonate formation temperature is 16°C using the equation of [68]Kim et al. (2007). This is the range of the average seawater temperature which varies between 13°C and 25°C at the site. Based on temperature measurements we can exclude that hydrothermal fluid is circulating and that aragonite precipitation is induced by elevated temperature.

In order to quantitatively demonstrate the contribution of AOM to the increase in carbonate saturation, we calculated saturation indices with respect to aragonite (Fig 8H), calcite and dolomite. These calculations reveal that dolomite (SI ≈ 2), calcite (SI ≈ 0.5) and aragonite (SI ≈ 0.5) are most supersaturated at the emission spots at the depths coincident with increased DIC and TA due to AOM (Fig 8). A decrease in Ca^2+^ of 2 mmol/l occurs at Emission Spot 2, probably as a result of CaCO_3_ precipitation, which apparently occurred faster than downward diffusion of Ca^2+^ from seawater. Increased TA resulting from AOM in addition to Ca^2+^ and Mg^2+^ ions from seawater near the sediment/water interface may induce cementation. Due to dynamic conditions of the gas conduits, the area and depth of precipitation may vary over time. This could explain why the TA and DIC maxima do not precisely coincide with the depth where the aragonite precipitates occur.

It is generally observed near the sediment-water interface that mostly aragonites are precipitated (e.g. [101]Bernoulli and McKenzie, 1981; [102]Burton, 1993), despite the fact that calcite and dolomite are the most supersaturated phases. It is also well known that dolomite formation is inhibited in seawater (e.g. [103]Land, 1998), and that the high Mg concentration of seawater inhibits the formation of calcite ([104]Berner, 1975). [105]Burton and Walter (1987) found that aragonite cements should be most abundant in warm to hot marine environments. Their experiments showed the temperature control of the precipitating carbonate mineral. Above 5°C aragonite precipitation rate is up to 5 times higher than calcite. The seawater temperature in the Tyrrhenian Sea does not fall below 12°C.

The crystal fan structure is characteristic for marine aragonite cement (e.g. [101]Bernoulli and McKenzie, 1981). A microbial role is often invoked to explain the spherical shapes of the precipitates (e.g. [106]Reitner et al., 2005; [107]Brauchli et al., 2013), but aragonite with exactly the same spherical fibrous structure can also form entirely abiotically in pure Ca-Mg-solutions (cf. [108]Fernández-Díaz et al., 2006; [43]Meister et al., 2011). Hence, it is not necessary to invoke any microbial effect other than an induced precipitation through TA increase by AOM. The spherical shape indicates that this phase is rapidly nucleating at relatively high supersaturation.

In conclusion, the aragonite cements observed at the Pomonte seep are induced by AOM activity, where the cements are spontaneously precipitated in the sediment as the interstitial solution becomes oversaturated. Furthermore, there is no indication of emerging hyperalkaline fluid (Fig 13D). This notion is supported by the negative carbon isotope values measured in the aragonite cements, which are not consistent with a Lost City-type hydrothermal vent carbonate. Because hyperalkaline fluid originating from serpentinization contains little DIC and carbonate is derived from seawater (or fresh water), carbonates related to serpentinization usually show δ^13^C near to DIC of seawater (cf. [109]Bernier et al., 1997; [36]Bach et al., 2011). We can explain the peculiar combination of abiotic CH_4_ seepage with microbially (AOM-) induced carbonate precipitation by the uncoupling of CH_4_ and CO_2_ (g) due to the absence of hydrothermally driven fluid flow at present, while the CH_4_ is still rising from greater depth.

## Conclusion

Methane seeps were observed offshore Pomonte, Elba Island, and adjacent islands of Pianosa and Scoglio d’Africa at shallow depth below the sea level. The CH_4_ at the Pomonte seep shows highest δ^2^H and δ^13^C values of -120‰ and -18‰, respectively, and δ^13^C values are inversely correlated with carbon number of hydrocarbon gases, which is consistent with an abiotic origin. In contrast, gas from Scoglio d’Africa could be of partial microbial origin.

The CH_4_ emerging from the Pomonte seep area is mostly likely of metamorphic origin, but it is unclear what role early high-temperature reactions versus present-day serpentinization reactions may play. By analogy with methane seepage in other ophiolites, a present-day generation of the methane appears perhaps more plausible. But the seep at Pomonte is different from other active serpentinization sites in that highly alkaline waters related to low-temperature serpentinization are not discharging. At the same time, δ^13^C_CH4_ values do not suggest extreme carbon limitation. This apparent decoupling of hydrothermal waters and a methane–rich gas phase may be due to segregation of gas and fluid due to a lack of a hydrothermal circulation.

Microbially mediated anaerobic methane oxidation occurs in the porous sand at the gas emission spots. Due to the lack of organic matter no other dissimilatory process occurs at significant rate. The high AOM activity increases the alkalinity in the porewater and induces the precipitation of aragonite cements with a spherical fibrous structure. Spherical aragonite needle aggregates typically form in seawater due to inhibition by Mg. In contrast, no influence of alkaline porefluid from the subsurface was noticed, as it commonly occurs at sites of serpentinization, which is consistent with the above-mentioned uncoupling of CH_4_ from its fluid. Instead a strongly adapted and specialized chemosynthetic microbial community has established, capable of efficiently harvesting the rising CH_4_ in a dynamic porewater system with changing conduits. The role of this type of abiogenic CH_4_-seep system in global biogeochemical cycles through Earth’s history and for life on the early Earth remains to be further evaluated.

## Supporting information

S1 Table Porewater chemistry and isotope compositions (δ^13^C_DIC_ and δ^18^O_H2O_) at gas emission and reference spots 1–3 (locations indicated on map; Fig 1). Samples were taken in 2011.
(XLSX)

S2 Table Additional sulphide and DIC concentrations and δ^13^C_DIC_-values of porewater samples taken in 2009 and measured in 2010. (XLSX)
